# Conjugated Polymer‐Microbe Interactions Trigger Regulatory Reprogramming for Enhanced Bioelectrocatalysis

**DOI:** 10.1002/advs.77927

**Published:** 2026-10-04

**Authors:** David Ohayon, Yuanmei Liang, Zexuan Wang, Jee Loon Foo, Glenn Quek, Samantha R. McCuskey, Zhongxin Chen, Matthew Wook Chang, Guillermo C. Bazan

**Affiliations:** ^1^ Institute For Functional Intelligent Materials National University of Singapore Singapore Singapore; ^2^ NUS Synthetic Biology for Clinical and Technological Innovation (SynCTI) National University of Singapore Singapore Singapore; ^3^ Synthetic Biology Translational Research Program Yong Loo Lin School of Medicine National University of Singapore Singapore Singapore; ^4^ Department of Biochemistry Yong Loo Lin School of Medicine National University of Singapore Singapore Singapore; ^5^ National Centre for Engineering Biology (NCEB) Singapore Singapore; ^6^ Departments of Chemistry and Chemical & Biomolecular Engineering National University of Singapore Singapore Singapore; ^7^ Institute For Digital Molecular Analytics and Science (IDMxS) Nanyang Technological University Singapore Singapore

**Keywords:** biochemistry, biophysics, cell biology, citric acid cycle, intracellular, microbial electrosynthesis, microbial fuel cell, redox, shewanella oneidensis

## Abstract

Abiotic redox‐active materials are increasingly used to interface with electroactive microorganisms, yet their influence on cellular regulation remains poorly understood. Here, *Shewanella oneidensis* MR‐1 was coupled with the electron‐transporting conjugated polymer poly(benzodifurandione) (PBFDO) to examine how material interfaces reshape inward extracellular electron transfer (EET). PBFDO‐coated carbon electrodes enhanced electron uptake and fumarate reduction, producing over two orders of magnitude higher current density and up to a twelvefold increase in succinate production than polymer‐free controls. Integrated electrochemical, transcriptomic, metabolomic, and protein analyses reveal coordinated physiological remodeling associated with the polymer‐supported interface rather than passive conductivity alone. PBFDO exposure is associated with transcriptional enrichment of genes linked to the Mtr‐CymA electron‐transfer conduit, energy metabolism, and biofilm formation. Metabolomics revealed altered intracellular redox and metabolite profiles consistent with enhanced fumarate respiration, alongside elevated NADH/NAD^+^ and ATP/ADP ratios indicative of altered cellular redox and energetic states. Beyond transcriptional regulation, polymer‐responsive small RNAs (sRNAs) revealed a post‐transcriptional regulatory layer linked to the biohybrid interface. Delivery of individual sRNAs into control cells partially recapitulated the polymer‐induced phenotype, increasing current output up to threefold while altering intracellular redox balance. These findings establish sRNA‐mediated regulation as a previously unrecognized mechanism linking biohybrid interfaces to enhanced microbial electrosynthesis.

## Graphical abstract

Conjugated polymer‐supported bioelectrochemical systems are shown to enhance microbial electrosynthesis by triggering coordinated electrochemical, transcriptional, post‐transcriptional (sRNA), metabolic, and protein‐level responses. Integrated multi‐omics and electrochemical analyses reveal selective physiological remodeling associated with improved interfacial electron transfer, providing a strategy for engineering more efficient bioelectrocatalytic systems.

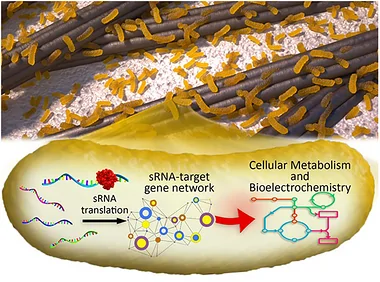

## 1 Introduction

Innovative strategies to effectively couple electrical inputs with biological catalysis offer promising routes for transitioning chemical synthesis and energy technologies toward sustainable, electrified platforms. For example, microbial electrochemical systems (MES) are increasingly recognized as versatile platforms to convert electrical energy into reduced chemicals using living cells as catalysts, combining enzymatic selectivity with the self‐repair and adaptability characteristics of microbial species [1, 2, 3]. These systems exploit the ability of certain microorganisms to exchange electrons directly with solid electrodes, thereby converting redox energy into cellular metabolism and vice versa. Such bioelectrochemical coupling has been proposed for diverse applications, ranging from wastewater treatment and carbon capture to sustainable production of biofuels and value‐added chemicals [2, 4, 5]. However, the performance of these systems can be constrained by the efficiency of electron exchange across the biofilm/electrode interface, which remains a central challenge for both outward electron transfer (current generation) and inward electron transfer (electrosynthesis) [6]. Beyond purely physicochemical considerations, it remains unclear whether the nature of the electrode interface itself can actively reshape microbial regulatory states and metabolic outputs.

Amongst known electroactive organisms, *Shewanella oneidensis* MR‐1 has emerged as a premier model for studying extracellular electron transfer (EET) because of its annotated genome and the detailed molecular characterization of its EET machinery, including the MtrCAB conduit and periplasmic fumarate reductases [7, 8, 9, 10]. Canonical EET in *S. oneidensis* is mediated by the multi‐heme cytochrome conduit MtrCAB, which spans the periplasm and outer membrane to transfer electrons to extracellular acceptors [11]. The organism also secretes flavins that act as soluble redox shuttles, supplementing direct electron transfer (DET) with mediated electron transfer (MET) pathways [12, 13, 14, 15]. Together, these mechanisms enable both outward electron flux and inward electron uptake for reductive metabolism [16, 17, 18]. Nevertheless, despite decades of genetic and biochemical study, the rate and efficiency of EET remain modest compared to abiotic catalysts, with limitations arising from slow cytochrome turnover, diffusional losses, limited cell–electrode contact area, and constrained supply of intracellular reducing equivalents [16, 19]. These bottlenecks have motivated strategies that combine biological and materials engineering to enhance the biofilm/electrode interface [20, 21].

Conductive organic polymers and conjugated polyelectrolytes offer a promising materials approach to bridge biochemical and electronic domains. These materials form soft conformal interfaces with tunable redox energetics and the potential to create percolated electroactive matrices that increase the number of cells in electronic communication with an external electrode [22, 23, 24]. Early studies with cationic or zwitterionic conjugated polyelectrolytes demonstrated that polymers can intimately coat bacteria and thereby enhance current generation [25, 26, 27, 28]. Mechanistic analyses further suggest that polymers modify local redox landscapes, alter mediator retention, and reduce interfacial impedance [29, 30, 31, 32]. Such effects suggest interactions that extend beyond passive conduction. More recently, n‐type conjugated polymers have been exploited as redox mediators since they can be molecularly engineered to exhibit redox potentials well‐matched to bacterial cytochromes, enabling both outward and inward EET [30, 31]. Most relevant to the present work, Zhu et al. demonstrated that a poly(benzodifurandione) (PBFDO) coating led to substantial improvements in anode performance and biofilm formation [33]. PBFDO formed a continuous film on carbon cloth, introduced multiple redox‐active features and pseudocapacitance, lowered the electrode charge transfer resistance, and yielded a ≈ 41% increase in power density, while enriching *Geobacter*‐dominated communities [33]. They further suggested that PBFDO participates in both direct and indirect electron transfer through its redox‐active backbone and hydrogen‐bonding interactions with flavins. However, mechanistic insights were focused on community‐level 16S sequencing and functional gene predictions, leaving unresolved whether polymer exposure directly reprograms bacterial regulatory networks and metabolism at the molecular level.

Despite progress on polymer–microbe composites, key mechanistic questions remain, particularly for inward EET. Most investigations have attributed performance improvements to increased electrode conductivity, pseudocapacitance, and surface hydrophilicity, which promote biomass attachment and mediator retention [29, 33, 34, 35, 36]. While these abiotic factors are undoubtedly important, recent transcriptomic and proteomic studies have begun to reveal that microbes may actively remodel their physiology in response to electrode environments [37, 38]. For instance, McCuskey et al. found through gene set enrichment analyses that blending *S. oneidensis* with a conjugated polyelectrolyte regulated NADH‐dependent metabolic pathways and induced the repression of motility genes, suggesting a shift toward surface‐associated lifestyles [38]. Those studies did not observe large changes in outer‐membrane cytochrome abundance, suggesting alternative regulatory layers beyond transcription. Such data remain scarce, are largely focused on outward EET or mixed communities, and rarely resolve molecular responses in single‐strain inward EET systems. At the same time, work on inward EET highlights that distinct gene sets underlie electron uptake and donation, emphasizing that polymer effects may differ depending on electrochemical directionality [12]. Critically, we are not aware of any studies to date that have connected polymer exposure to post‐transcriptional regulation, despite the central role of such mechanisms in rapid bacterial adaptation.

Small, non‐coding RNAs (sRNAs) are central post‐transcriptional regulators that modulate mRNA stability and translation, coordinate stress responses, and rewire metabolism [39, 40]. They act through base‐pairing interactions with target mRNAs and recruitment of RNA chaperones, enabling rapid, multifactorial responses to environmental changes. sRNAs are enriched for targets involved in membrane composition, transport, and redox‐associated processes, functional domains that directly shape EET by controlling envelope structure and respiratory network assembly. In *S. oneidensis*, the iron‐responsive sRNA RyhB modulates expression of multiple c‐type cytochromes, demonstrating direct sRNA control of EET‐relevant machinery. Engineered CRISPRi–sRNA constructs targeting mtr genes further alter electrochemical outputs [41, 42]. Broader surveys in other exoelectrogens (e.g., *Geobacter soli*) have likewise identified differentially expressed sRNAs under different electron acceptor conditions [43].

Despite these leads, systematic investigations of sRNA function in MES remain limited. Few studies combine sRNA discovery with direct electrochemical phenotyping, and, to the best of our knowledge, none has examined whether the presence of conductive polymers triggers specific sRNA‐mediated regulation. At the same time, synthetic sRNAs are increasingly recognized as tractable tools to tune gene expression, biofilm formation, and metabolic flux [40, 42]. The scarcity of sRNA studies in MES opens questions about their role in mediating bacterial adaptation to different electrode interfaces and their potential to be harnessed for reprogramming biofilms to enhance electrosynthesis. To our knowledge, no prior work has linked conjugated polymer exposure to sRNA expression. This gap represents a critical frontier in determining whether engineered materials can reprogram bacterial function through sRNA signaling, opening a new dimension of polymer–microbe biohybrids in which abiotic interfaces control not only electron transfer but also regulatory networks that dictate cellular physiology. Conceptually, we consider a causal hierarchy in which the material interface defines the local redox environment sensed by the cell, triggering regulatory reprogramming that ultimately reshapes metabolic flux and electrochemical output. Disentangling these levels, from interfacial chemistry to post‐transcriptional control, is essential to determine whether conductive polymers act merely as electronic conduits or as instructive biological interfaces.

To address these opportunities, we selected the n‐type conjugated polymer PBFDO due to its previously established conductivity, strong pseudocapacitance, and a redox window well aligned with bacterial electron uptake [33, 44]. Using *S. oneidensis* fumarate reduction as a model inward EET reaction, we show that PBFDO markedly increases steady‐state current density, succinate production, biomass accumulation, and current per cell. To probe underlying mechanisms, we combined electrochemical measurements with transcriptomic, metabolomic, and sRNA analysis. Transcriptomics revealed coordinated upregulation of redox and energy metabolism, enhanced biofilm‐associated programs, and downregulation of membrane protein folding factors. Complementary metabolomic analysis revealed changes in intracellular metabolite abundance consistent with enhanced fumarate reduction and altered electron allocation. Most strikingly, PBFDO exposure was accompanied by the induction of multiple sRNAs. Bioinformatic predictions of synthetic sRNAs indicated regulatory links to envelope remodeling and redox metabolism, and experimental delivery of selected candidates reproducibly enhanced both current and succinate production. These results establish a causal hierarchy in which the polymer interface reshapes cellular redox states, activates sRNA‐mediated post‐transcriptional regulation, and ultimately enhances metabolic and electrochemical outputs, revealing a previously unrecognized regulatory axis in polymer–microbe biohybrids.

## 2 Results and Discussion

Electron‐transporting n‐type materials are particularly attractive for electron injection into electroactive bacteria, provided their redox potential and conductive window align with the bacterial reduction potential. To evaluate these effects in a controlled setting, we first examined how PBFDO influences bioelectrochemical performance in *S. oneidensis* reactors. As a model for inward extracellular EET process, we focused on the two‐electron reduction of fumarate to succinate, which is catalysed by periplasmic fumarate reductases receiving electrons from the electrode via the Mtr conduit (Figure 1a) [45]. This well‐defined pathway provides a controlled platform to quantify conjugated polymer effects on electron injection and metabolism.

**FIGURE 1:**
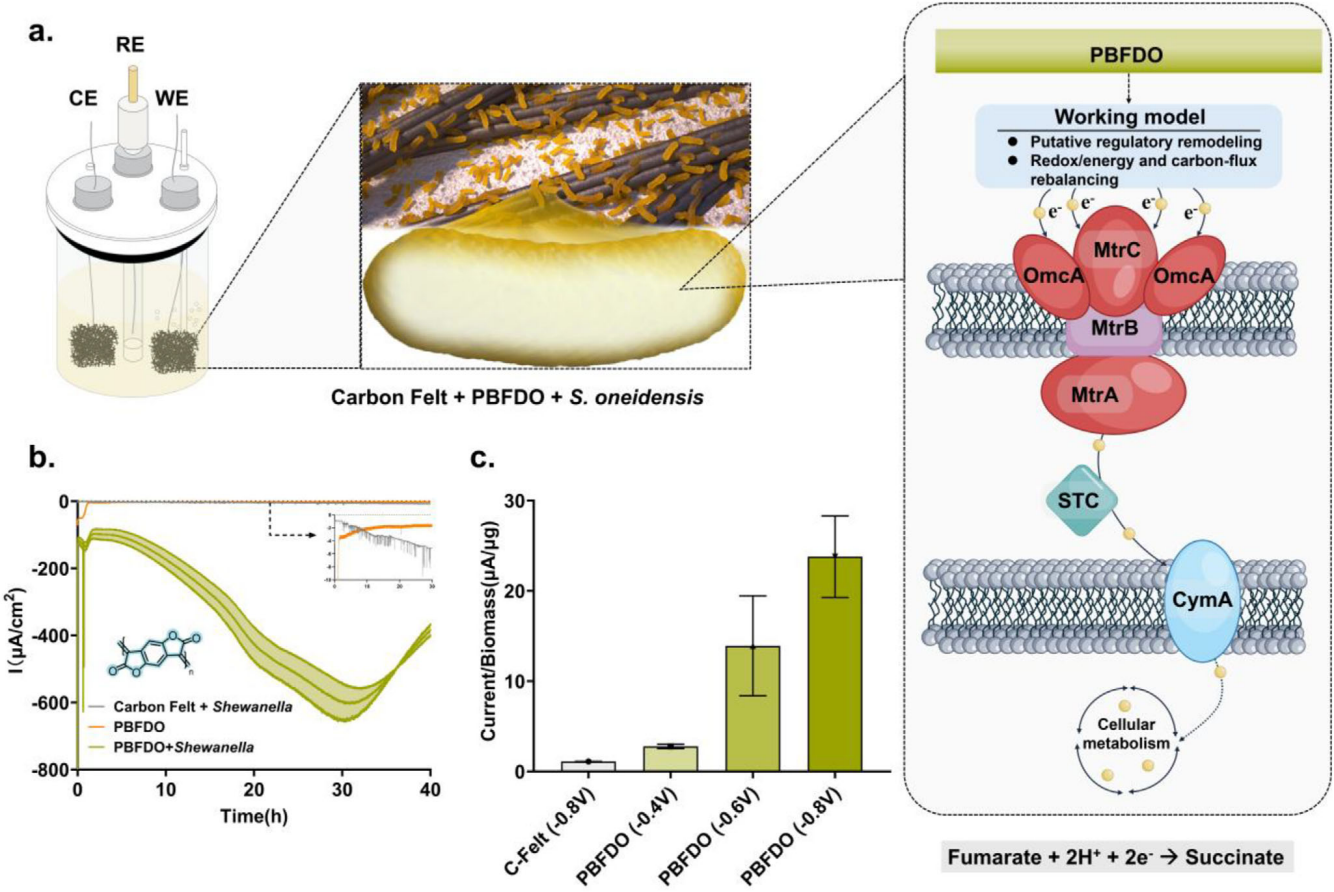
(a) *Shewanella oneidensis* is coupled with the n‐type polymer PBFDO to build a living bioelectrochemical microbial composite. Representative scanning electron microscopy images of the PBFDO‐coated electrodes with *S. oneidensis*. Proposed model for the extracellular electron uptake by *S. oneidensis* from the electrode through its Mtr respiratory pathway; b. Current as a function of time for control (carbon felt electrode with *S. oneidensis*), biocomposite (PBFDO polymer‐coated carbon felt electrode coated with *S. oneidensis*), and negative control (carbon felt electrode with PBFDO polymer). The electrochemical cells were poised at ‐0.8 V vs. Ag/AgCl (N = 3), and 50 mM fumarate was added at t = 0 h. c. Current per attached biomass extracted from bioelectrochemical reactors poised at different potentials. Biomass was extracted from the bacteria attached to the bioreactor working electrodes and quantified through a Bradford assay.

PBFDO was deposited as a thin conformal coating on carbon felt electrodes prior to bacterial inoculation (see Methods). When PBFDO‐coated electrodes were paired with *S. oneidensis* in a three‐electrode configuration, we observed significant improvements in both electrochemical and metabolic performance compared to uncoated controls. At an applied potential of –0.8 V vs. Ag/AgCl, PBFDO‐supported biocomposites achieved a maximum 130‐fold increase in current density (696.25 ± 110.42 µA/cm^2^ vs 5.44 ± 0.29 µA/cm^2^ for the control, see Figure 1b). Negative controls (polymer‐coated electrodes only) exhibited negligible cathodic currents, confirming that current generation originated from bacterial activity. The enhanced current was accompanied by a 10‐fold increase in succinate production (21.47 ± 0.60 vs 2.26 ± 0.19 mM; Figure S1), demonstrating that PBFDO improves both charge transfer and enzymatic conversion efficiency.

Biomass quantification revealed 5–10× greater colonization on PBFDO electrodes, consistent with prior reports that conjugated polymers promote bacterial adhesion and biofilm development (Figure S2). SEM imaging confirmed substantially greater surface colonization and biofilm coverage on PBFDO‐coated electrodes compared with the sparse, patchy colonization observed on bare carbon‐felt electrodes (Figure S3). Gross cellular morphology remained comparable between conditions, indicating that PBFDO primarily promotes biofilm establishment rather than altering cell morphology. While increased biomass partly explains the higher currents, normalization of current density to attached biomass revealed that PBFDO systems still exhibited superior per‐cell electron transfer efficiency, indicating that the polymer interface enhances intrinsic cellular electron uptake beyond its effects on attachment (Figure 1c). The gain in electrochemical output, therefore, cannot be attributed solely to increased surface colonization.

We further probed how performance varied with electrode potential. We investigated the electron injection efficiency at multiple applied potentials (−0.4, −0.6, and −0.8 V vs. Ag/AgCl). Across all conditions, PBFDO‐based reactors consistently outperformed controls (Figure 1c). Even at ‐0.4 V, PBFDO‐enhanced cultures generated substantially higher current densities and supported greater biomass accumulation (Figure S2). For downstream transcriptomic and metabolomic analyses, −0.6 V was selected as a compromise potential that provided robust inward electron transfer while minimizing potential‐dependent physicochemical stress and non‐biological side reactions. Overall, these results indicate that the effect of PBFDO persists across physiologically relevant redox regimes [46, 47, 48].

Electrochemical characterization by cyclic voltammetry (CV) and differential pulse voltammetry (DPV) provided additional insight into electron‐transfer pathways operating under PBFDO exposure (Figure S4). Measurements performed immediately after reactor operation revealed enhanced cathodic catalytic currents in PBFDO‐associated biofilms relative to carbon‐felt controls (Figure S4a). To distinguish electrode‐associated and soluble redox contributions, colonized electrodes were subsequently rinsed and transferred into fresh degassed medium, while spent supernatants were analyzed separately using a clean glassy carbon electrode. DPV measurements of PBFDO‐supported colonized electrodes revealed a redox feature centered near +0.09 V vs. Ag/AgCl, consistent with outer‐membrane c‐type cytochromes involved in extracellular electron transfer, together with a second feature near −0.10 V that is consistent with cytochrome‐bound flavins (Figure S4d) [49, 50, 51]. These signals remained detectable after electrode rinsing, indicating persistence of electrode‐associated redox activity. In contrast, the broad catalytic cathodic response observed by CV was substantially diminished after transfer into fresh medium but reappeared upon analysis of spent supernatants, demonstrating a contribution from soluble redox‐active species accumulated during reactor operation (Figure S4a‐e). A similar trend was observed for control bioreactors. Furthermore, the analysis of spent medium further revealed a reproducible soluble redox feature near −0.62 V vs. Ag/AgCl in both PBFDO‐supported and carbon‐felt systems (Figure S4f). Because this signal was present under both conditions at nearly identical potentials, it is unlikely to originate from polymer‐derived products or polymer leaching. The comparatively modest increase in soluble redox signals relative to the substantially larger increase in electrode‐associated catalytic currents further indicates that soluble mediators alone cannot account for the enhanced electron uptake observed in PBFDO‐supported reactors. Together, these results support contributions from both electrode‐associated and mediator‐assisted electron transfer and indicate that PBFDO enhances native electron‐transfer pathways rather than introducing unique polymer‐derived redox mediators.

Biocompatibility was assessed using live/dead staining and confocal laser scanning microscopy following electrochemical operation. Live (SYTO 9, green) and dead (propidium iodide, red) staining revealed minimal cell death in both PBFDO‐based (93.5% cells viable) and control biofilms (99.2% cells viable) (Figure S5), indicating that PBFDO is non‐toxic to the bacteria under electrochemical operation. These data establish PBFDO as a biocompatible, high‐performance interface that enhances inward EET and fumarate reduction in *S. oneidensis*. However, the magnitude of the enhancement exceeds what can be explained by increased biomass or passive conductivity alone, motivating investigation of whether the polymer interface might induce deeper physiological and regulatory adaptation.

To investigate whether PBFDO exposure alters *S. oneidensis* physiology beyond passive enhancement of electron transfer, we performed transcriptomic and metabolomic analyses of electrode‐associated biofilms following steady‐state current collection. RNA sequencing revealed distinct pathway‐coherent transcriptional changes in *S. oneidensis* cells exposed to PBFDO compared to control conditions (Figure 2, Table S1a and Supplementary Discussion S1). Rather than acting solely as a conductive scaffold, PBFDO induced a reproducible transcriptional program distinct from carbon electrode controls. Transcriptomic profiling reveals a targeted response rather than a global shift. PBFDO and control replicates segregate by condition in hierarchical clustering (Figure 2a), while most transcripts remain unchanged (Figure 2b). Using adjusted p‐value thresholds (padj < 0.05) and |log2FC| > 1, we identified 405 differentially expressed genes out of 4, 034 detected transcripts, comprising 372 upregulated and 33 downregulated genes under PBFDO exposure (Figure 2b). Upregulated genes were enriched in electron transfer, energy metabolism, and biofilm‐related functions, whereas a smaller subset of genes involved in motility and chemotaxis were downregulated. Upregulated genes included electron‐transfer components (*fccA*, *ompA*, *STC*), ribosomal proteins, and biofilm adhesin *bpfA*, alongside iron‐regulatory factors (*fur*, *iscR*) and ferredoxin (*fdx*). Downregulated genes were enriched in chemotaxis signaling (*cheD*, methyl‐accepting chemotaxis proteins) and flagellar motor components (*pomA*, *fliG*), consistent with a transition from a motile to a surface‐attached state despite concurrent upregulation of flagellar structural genes. This coordinated response indicates coordinated modulation across respiratory modules [52, 53]. This is supported by the elevated intracellular NADH/NAD^+^ ratio under PBFDO, consistent with increased intracellular reductant availability [54, 55]. PBFDO exposure also shifted expression toward a more surface‐associated biofilm state (adhesion/biofilm genes up, motility/chemotaxis down), consistent with enhanced attachment on PBFDO‐coated electrodes (Table S1a; Supplementary Discussion S1).

**FIGURE 2:**
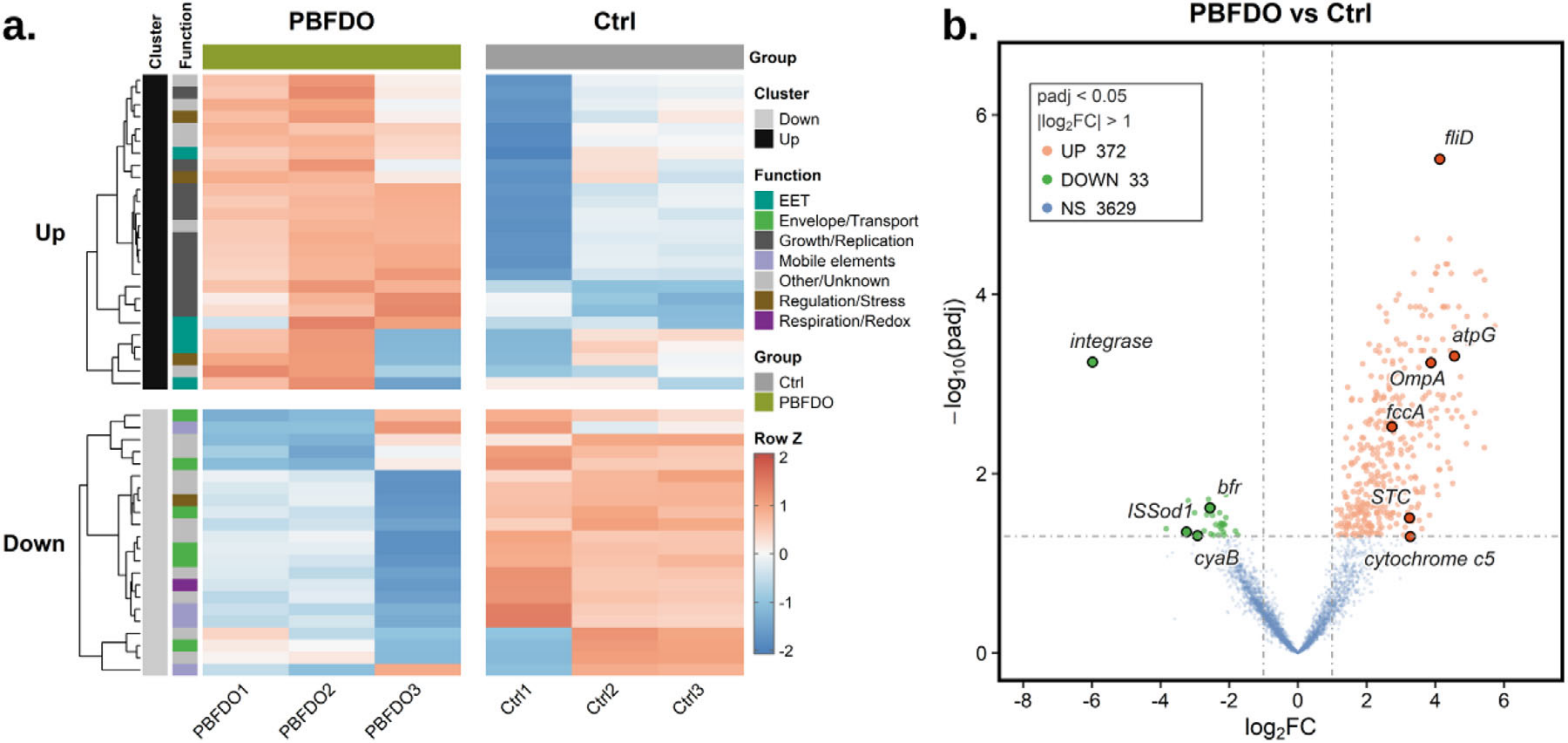
a. The clustering heat map of 6 samples based on all the differentially expressed genes of *S. oneidensis* across 2 groups (untreated control on carbon electrodes and coupled with PBFDO‐coated carbon electrodes); b. Volcano plot of statistical significance (−log_10_ p‐value) against enrichment (log_2_ fold change) of differentially expressed genes of *S. oneidensis* in control (without polymer) and PBFDO‐coated electrodes. Total numbers of differentially expressed genes are shown in brackets. Upregulated genes are presented in orange (UP, log2FC>1), while downregulated genes are presented in green (DOWN, |log2FC|>1). Non‐significantly differentially expressed genes are shown in light blue (NS, ‐1<log2FC<1).

These observations parallel prior analyses of *S. oneidensis* interfaced with conductive polymers, where metabolic modules supplying reducing equivalents were preferentially modulated [38]. This suggests a bias toward maximizing intracellular reducing power and coupling it efficiently to terminal electron‐transfer pathways, independent of electron‐flow direction. However, the specific regulatory targets differ between systems, indicating that polymer chemistry and electrochemical context shape distinct physiological adaptations.

Building on the transcriptomic analysis, we examined which respiratory and energy‐converting modules were most responsive to PBFDO exposure. Targeted qPCR confirmed increased transcript abundance across components of the conduit–energy axis (*mtr*/*cymA* → *nuo* → *atp*), together with elevated *fccA* transcript levels, consistent with enhanced periplasmic fumarate‐reduction capacity (Figures 3a–c, Table S2). Consistent with these transcriptional changes, heme‐stained protein gels revealed increased abundance of heme‐containing proteins in PBFDO‐supported biofilms relative to carbon‐felt controls (Figure 3d). Transcript abundance of *mtrC* and *cymA* positively correlated with biomass‐normalized current, supporting enhanced conduit efficiency as a contributor to increased electron throughput per cell [47, 56]. Elevated transcript levels of Complex I subunits (*nuoF*, *nuoL*) and ATP synthase (*atpD*) are consistent with transcriptional reorganization of energy‐conversion and redox‐balancing pathways that may support increased electron influx (Figure 3b) [57].

**FIGURE 3:**
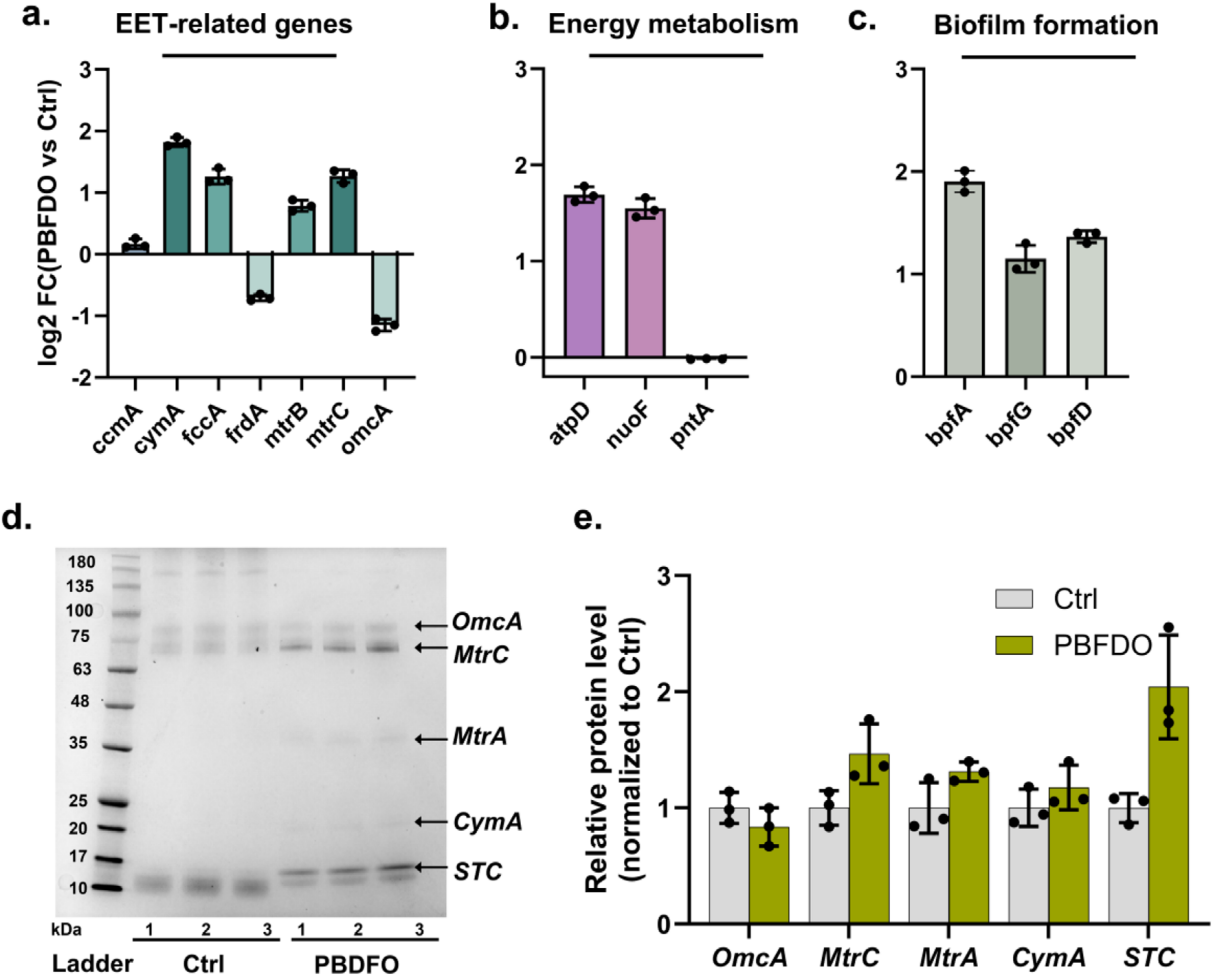
a. EET‐related genes. qPCR log_2_ fold‐changes (PBFDO vs Control) for *ccmA*, *cymA*, *fccA*, *frdA*, *mtrB*, *mtrC*, and *omcA*; b. Energy metabolism. qPCR log_2_ FC for *atpD*, *nuoF*, and *pntA*; c. Biofilm formation. qPCR log_2_ FC for *bpfA*, *bpfG*, and *bpfD*. In panels (a–c), bars denote mean ± SEM, and overlaid dots are biological replicates (n shown by points). Fold‐changes were calculated relative to the control condition using the geometric mean of two housekeeping genes for normalization; d. Representative heme staining of c‐type cytochromes in *S. oneidensis* MR‐1 biofilms grown on carbon‐felt (Ctrl) or PBFDO‐coated electrodes (PBFDO). Heme‐containing proteins detected include OmcA, MtrC, MtrA, CymA, and STC; e. Relative protein levels of OmcA, MtrC, MtrA, CymA, and STC in PBFDO‐coated groups normalized to the Ctrl condition. Data represent mean ± SD of three biological replicates (n = 3).

PBFDO also triggered activation of the *bpfA*/*bpfG*/*bpfD* operon, encoding the RTX‐type adhesin *BpfA* and its secretion machinery (Figure 3c). Prior studies identify *BpfA* as a major determinant of biofilm integrity [58]. Consistent with this program, electrode‐associated biomass increased approximately tenfold relative to the polymer‐free control (Figure S2). Genes comprising the conduit–energy axis clustered closely along the identity line, supporting strong agreement between RNA‐seq and qPCR measurements [59]. Together, these data identify a coordinated transcriptional state associated with PBFDO‐supported inward electron transfer, encompassing respiratory, energy‐conversion, and biofilm‐associated pathways. Increased adhesion contributes to biomass accumulation, while changes in conduit‐ and energy‐related gene expression are consistent with enhanced electron uptake and electrosynthetic performance [56]. The observation of increased heme‐containing protein abundance in PBFDO‐supported biofilms further supports the involvement of cytochrome‐associated electron‐transfer processes. To further evaluate EET machinery at the protein level, we performed heme staining to visualize c‐type cytochrome abundance in biofilm lysates. Heme‐containing proteins, including MtrC, MtrA, CymA, STC, and OmcA, were detected in both conditions (Figures 3d, e). Quantification revealed increased abundance of STC and MtrC under PBFDO conditions, with more modest increases in MtrA and CymA, whereas OmcA remained largely unchanged. The maintenance of c‐type cytochrome pools under PBFDO conditions indicates that the conjugated polymer does not interfere with heme biosynthesis or cytochrome maturation. These observations broadly mirror the transcriptional trends and indicate that PBFDO‐supported biofilms undergo selective remodeling of cytochrome‐associated electron‐transfer machinery while maintaining overall c‐type cytochrome maturation. We also note that transcriptomic analyses were performed using equivalent total RNA inputs and standard library‐size normalization rather than normalization to cell number. Consequently, while the differential expression analysis compares transcriptome composition between conditions, it does not directly quantify transcript abundance on a per‐cell basis. Accordingly, a contribution of differences in biofilm biomass to the observed transcriptional profiles cannot be completely excluded. However, the consistency between the transcriptomic results, DNA‐normalized metabolomics, biomass‐normalized electrochemical measurements, and heme‐staining analyses supports the conclusion that the observed molecular differences are not solely attributable to increased biofilm accumulation and are consistent with genuine physiological remodeling associated with PBFDO‐supported inward electron transfer.

To examine how these transcriptional changes were associated with metabolic outcomes, we quantified fumarate consumption, succinate production, and the fraction of substrate conversion recovered in the measured product (Figure 4). We also compared intracellular redox and energy‐related metabolite ratios between conditions. To further examine the relationship between substrate consumption and product formation, we performed partial substrate–product carbon accounting under the −0.6 V conditions used for the mechanistic analyses. Importantly, because fumarate was the only metabolizable exogenous carbon source present during electrochemical operation, changes in fumarate consumption and succinate formation directly reflect alterations in respiratory substrate utilization under inward electron‐transfer conditions. Fumarate decreased from 50 mM to 38.3 ± 2.6 mM in the carbon‐felt controls and to 17.7 ± 3.4 mM in the PBFDO‐supported reactors, while succinate accumulated to 2.26 ± 0.19 and 21.47 ± 0.60 mM, respectively. Based on the group‐mean concentrations, approximately 19% of the consumed fumarate carbon was recovered as succinate in the control, compared with approximately 67% under PBFDO‐supported conditions. This corresponds to an approximately 3.5‐fold increase in the recovery of consumed fumarate as succinate relative to the carbon‐felt control (Table 1). Because biomass carbon, extracellular polymeric substances, additional intracellular and extracellular metabolites, and inorganic carbon (CO_2_) were not comprehensively quantified, this analysis represents partial substrate–product carbon accounting rather than a fully closed carbon balance.

**FIGURE 4:**
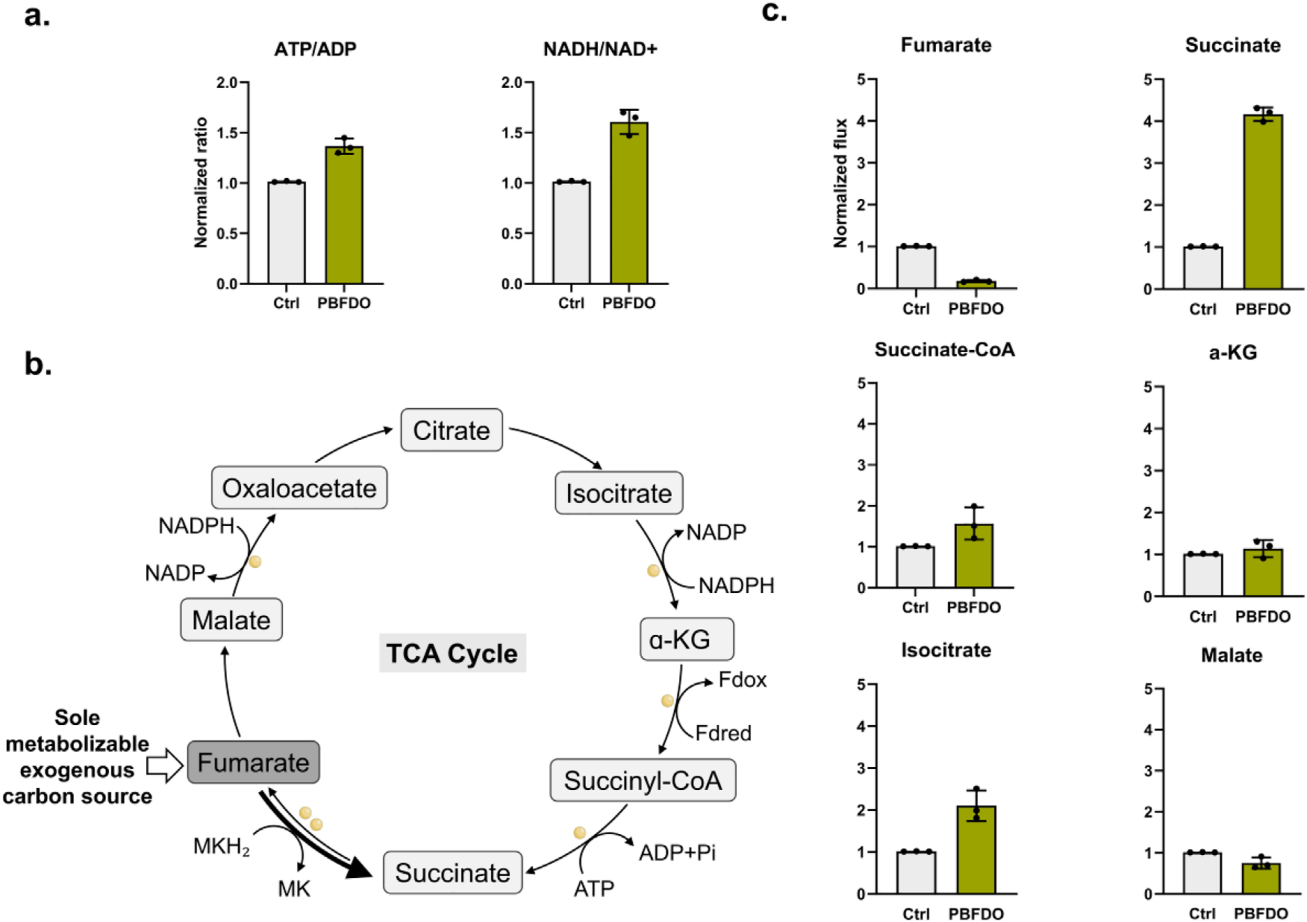
Untargeted metabolomic responses to PBFDO‐coated electrodes in *Shewanella oneidensis* MR‐1. a. Intracellular energy and redox ratios under control and PBFDO conditions, showing changes in the ATP/ADP and NADH/NAD^+^ ratios; b. Schematic overview of the tricarboxylic acid (TCA) cycle, highlighting the metabolites quantified in this study. Fumarate reduction to succinate is mediated via menaquinol (MKH_2_) as the electron donor (MKH_2_/MK), consistent with the periplasmic FccA‐dependent respiratory pathway in *S. oneidensis*. c. Relative levels of key TCA‐cycle intermediates under control and PBFDO conditions, including fumarate, succinate, succinyl‐CoA, α‐ketoglutarate (α‐KG), isocitrate, and malate. Data are presented as normalized values relative to the control group and shown as mean ± SD (*n* = 3biological replicates).

**TABLE 1:** Electrochemical performance, succinate production, and substrate‐to‐product conversion metrics for PBFDO‐supported and control bioreactors.

	Q (C)	n_e (mmol e^−^)	[Succinate] (mM)	n_e_succ_ (mmol e^−^)
Control	1.88 ± 0.83	0.019 ± 0.001	2.26 ± 0.19	0.072 ± 0.006
PBFDO	201.03 ± 80.69	2.08 ± 0.84	21.47 ± 0.60	0.687 ± 0.019
Net (PBFDO‐Ctrl)	199.15 ± 0.83	2.06 ± 0.83	—	0.615 ± 0.02
CE	29.77% ± 12.10%			
EPI	3.36 ± 1.37			
SRI	3.5			

Q: integrated charge from current trace; n_e: moles of electrons from current trace; n_e_succ_: moles of electrons involved in succinate production; CE: Coulombic Efficiency; EPI: Electron Partition Index; SRI: Succinate Recovery Index (ratio of the fraction of consumed fumarate recovered as succinate in PBFDO‐supported reactors relative to carbon‐felt controls).

Table 1 summarizes the distribution of cathodic electrons among measurable sinks. PBFDO exposure markedly increases total charge uptake (Δn_e_ ≈ 2.06 mmol e^−^ vs. 0.019 mmol e^−^ in control) and succinate accumulation (Δn_e_succ ≈ 0.687 mmol e^−^ vs. 0.072 mmol e^−^ in control), yielding a Coulombic efficiency (CE) of ∼30% and an Electron Partition Index (EPI) of ∼3.4 (ratio of total electron uptake to electrons recovered in succinate). These metrics indicate that a fraction of the additional electrode‐derived electrons was recovered in succinate, while the remaining electrons were not quantitatively resolved. The elevated NADH/NAD^+^ and ATP/ADP ratios are consistent with altered cellular redox and energetic states, but do not quantitatively account for the unrecovered electrons (Figure 4a). This allocation pattern aligns with systems‐level models in which *S. oneidensis* flexibly channels electrons across periplasmic and cytoplasmic branches according to interfacial and energetic demands [52].

At the network level, untargeted metabolomics revealed changes in metabolite abundance associated with fumarate reduction and intracellular redox balancing (Figure 4b,c), mirroring the CE‐EPI relationship. Such partitioning is characteristic of bioelectrochemical systems, where product‐based CE alone underestimates the physiological use of electrode‐derived electrons. Within this framework, EPI complements CE by quantifying the extent to which total electron flux exceeds the measured product yield, quantifying total electron flux relative to product yield [60]. Because both PBFDO‐coated and bare electrodes impose inward EET conditions and support direct cell–electrode contact, the observed metabolic divergence cannot be attributed to electron uptake alone. Instead, the observed metabolic divergence is consistent with changes in the local interfacial redox environment associated with PBFDO‐supported inward electron transfer, which may stabilize electron delivery into the quinone and periplasmic redox networks and thereby reshape downstream electron allocation. This interpretation is consistent with transcriptional enrichment of components of the CymA–MtrCAB conduit and associated periplasmic redox relays, together with increased abundance of heme‐containing proteins detected in PBFDO‐supported biofilms [47]. Endogenous flavins and other secreted redox‐active metabolites can contribute to electron exchange at the biofilm–electrode interface (Figure S6). PBFDO likely interacts with this native redox environment and may facilitate electron transfer through combined effects on interfacial redox chemistry, utilization of endogenous mediators, electron delivery, and cellular physiology. Electrochemical analysis of spent supernatants revealed soluble redox‐active species under both PBFDO and carbon‐felt conditions, arguing against polymer‐derived mediators or significant polymer leaching. Consistent with these measurements, metabolomic analysis showed an approximately two‐fold increase in extracellular riboflavin abundance under PBFDO‐supported conditions, whereas FMN and FAD levels remained largely unchanged (Figure S6). Although these results indicate a modest enhancement in endogenous soluble redox species, the increase is substantially smaller than the orders‐of‐magnitude enhancement in electrode‐associated catalytic currents. Together, these observations suggest that PBFDO primarily improves interfacial electron transfer while also promoting more effective utilization of native soluble mediator networks. This response was accompanied by elevated NADH/NAD^+^ and ATP/ADP ratios, consistent with altered intracellular redox and energetic states [13].

The branched architecture of the periplasmic redox network suggests a framework for reconciling increased succinate production with broader metabolic reprogramming [52, 61]. Strengthening of periplasmic electron relays under PBFDO exposure enhances fumarate respiration without requiring proportional changes across all inner‐membrane components. Collectively, increased succinate production, elevated NADH/NAD^+^, and ATP/ADP ratios, and the CE–EPI balance support a model in which cathodic electrons enter the quinone and periplasmic networks through the CymA–MtrCAB conduit and are partitioned between fumarate reduction and intracellular energy conservation. This periplasm‐centered electron‐transfer regime, reinforced by flavin‐mediated exchange and the conductive PBFDO interface, represents a highly efficient mode of inward EET in *S. oneidensis* [53, 62]. Taken together, these multi‐layered changes suggest that PBFDO exposure does not simply enhance electron flux but is associated with coordinated transcriptional responses involving respiratory, energy‐conversion, and biofilm‐associated pathways. We therefore asked whether post‐transcriptional control by small RNAs acts as a central mediator of this polymer‐induced reprogramming.

Small non‐coding RNAs are well‐established, fast‐acting regulators in Gram‐negative bacteria that modulate mRNA stability and translation, tuning membrane composition, transport, and metabolism. Because the coordinated transcriptional and metabolic state induced by PBFDO is unlikely to arise from transcriptional control alone, we next tested whether post‐transcriptional regulation by small RNAs constitutes a mechanistic layer in this polymer‐induced physiological program. To address this, we implemented a systematic pipeline to reconstruct and predict candidate PBFDO‐responsive sRNAs from RNA‐seq data collected directly from polymer‐coupled reactors (Figure S7). This workflow combined read assembly, secondary‐structure modelling, and target prediction (IntaRNA), followed by functional categorization of likely mRNA targets.

Five high‐confidence sRNA candidates (sRNA_001 to sRNA_005, Table S3) were reproducibly enriched under PBFDO conditions. Notably, these sRNAs were induced specifically under polymer‐coupled conditions, positioning them as candidate regulatory mediators linking interfacial redox cues to intracellular reprogramming. These sequences represent the five highest‐confidence reconstructed sRNAs based on read abundance, structural stability, and reproducibility across biological replicates, and were prioritized as putative regulators of the polymer‐induced state. Predicted targets converge on functions relevant to electron transfer and surface interactions, including DMT‐family transporters, the Trk potassium uptake system, cytochrome c biogenesis factors (*Ccm*/*Ccm1*‐type), formate dehydrogenase maturation factor *FdhD*, the Fdh β‐subunit, *MurB* (UDP‐N‐acetylmuramate dehydrogenase), and the *YedE*/*FdrT* membrane protein family, as well as enzymes implicated in selenium handling (Figure 5a). These targets converge on membrane assembly, ion and metal transport, and redox enzyme maturation, all functional nodes that directly shape periplasmic electron routing and cell–surface coupling. Predicted regulation of *MurB* and *FdhD* provides plausible links between envelope remodeling, redox enzyme maturation, and the observed adhesion and metabolic phenotypes. Importantly, we note that sRNAs typically have multiple targets; thus, the predicted interactions likely reflect distributed regulatory effects rather than single‐gene control. The molecular targets inferred from computational prediction await direct experimental validation. Future studies employing targeted qPCR, translational reporter assays, and complementary genetic approaches will be required to confirm individual sRNA–mRNA interactions and establish the precise post‐transcriptional mechanisms by which these sRNAs regulate electron‐transfer efficiency.

**FIGURE 5:**
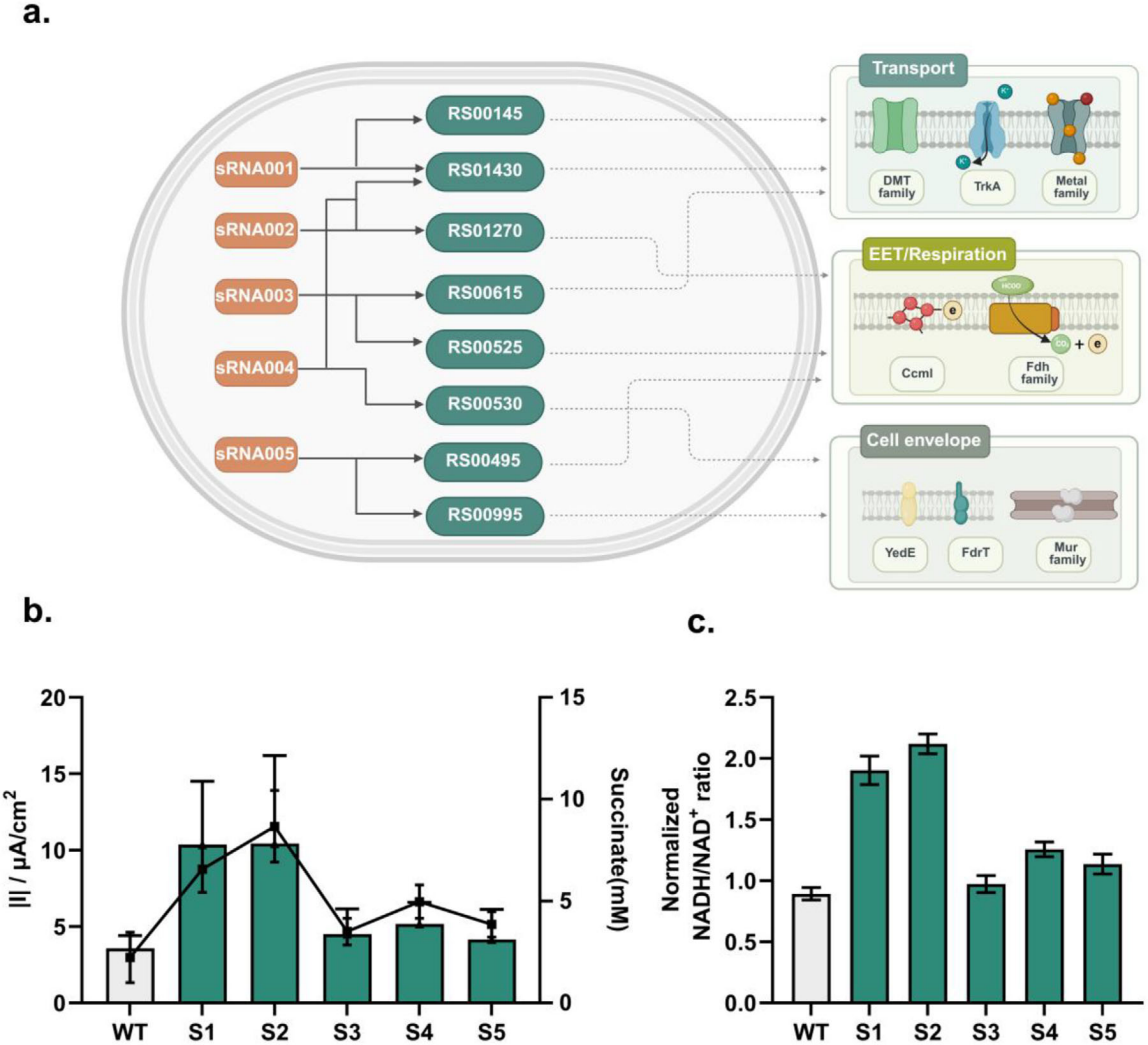
a. Schematic summary of the predicted sRNA–target gene network and their associated functional categories. Target genes were grouped into three major modules, including transport, EET/respiration, and cell envelope functions. b. Average current density (bars, left y‐axis) and extracellular succinate concentration (line with error bars, right y‐axis) obtained from microbial reactors containing sRNA‐modified *S. oneidensis* vs control; c. Associated normalized NADH/NAD^+^ ratio obtained from assay. Data are presented as mean ± SD (n = 3 biological replicates).

To validate these predictions, we expressed each candidate sRNA individually in *S. oneidensis* using plasmid‐based delivery and evaluated their effects in polymer‐free bioelectrochemical reactors. Each sRNA‐expressing strain (*S. oneidensis* + sRNA_i) was compared against a WT control processed in parallel under identical electrochemical conditions (Figure 5b). Although individual sRNA expression cannot fully recapitulate the polymer‐induced state, this controlled perturbation strategy tests whether discrete post‐transcriptional regulators are sufficient to reproduce key electrochemical and metabolic phenotypes.

The effects were sequence‐dependent and reproducible across biological replicates. Expression of sRNA_001 (predicted to affect a DMT transporter and Trk potassium uptake) and sRNA_002 (predicted to affect a DMT transporter and cytochrome‐c biogenesis factor Ccm1) produced the largest gains in steady‐state current, with I/I(WT) ≈ 2.9 for both constructs. sRNA_004 (DMT transporter, YedE/FdrT, selenium metabolism) and sRNA_003 (metal transporter, FdhD) yielded intermediate increases (I/I(WT) ≈ 1.45 and 1.26, respectively), while sRNA_005 (Fdh β‐subunit, MurB) produced a modest effect (I/I(WT) ≈ 1.17). Importantly, induction of single sRNAs in the absence of polymer was sufficient to enhance electron uptake, demonstrating that post‐transcriptional programs alone can modulate inward EET efficiency.

Extracellular succinate production was concomitantly elevated across all five sRNA‐overexpressing strains. sRNA_001 and sRNA_002 exhibited the largest increases (∼3.0‐ and ∼3.9‐fold relative to WT, respectively), closely mirroring their current density enhancements (∼2.9‐fold each), whereas sRNA_003–sRNA_005 showed more moderate increases in succinate production (1.6–2.2‐fold), consistent with their intermediate electrochemical phenotypes (Figure 5b). Together with the elevated NADH/NAD^+^ ratios, these results support a link between sRNA‐mediated regulation, intracellular redox balance, and enhanced electrosynthetic fumarate reduction. Most sRNA treatments also showed modest increases in NADH/NAD^+^ ratios after current collection, consistent with an expanded intracellular reducing pool (Figure 5c). SEM revealed sRNA‐dependent differences in biofilm architectures: sRNA_003 produced thick, multilayered films; sRNA_005 induced more discrete island clusters; sRNA_001, sRNA_002, and sRNA_004 produced biofilm patterns similar to, or slightly denser than, WT (Figure S8). These morphological differences are interpreted here as downstream phenotypic manifestations of individual regulatory perturbations rather than as direct evidence of the mechanisms responsible for enhanced electron uptake.

Notably, *S. oneidensis* on bare carbon felt does not spontaneously activate this regulatory program, despite experiencing inward EET and direct electrode contact. This observation underscores that polymer exposure provides a distinct interfacial and redox context (through altered charge‐transfer kinetics, redox buffering, and surface chemistry) that stabilizes a physiological state conducive to sRNA induction. In this framework, the PBFDO‐supported inward EET state acts as the upstream stimulus, defined by its interfacial chemistry and redox properties, while sRNAs function downstream as regulatory effectors that translate interfacial redox cues into coordinated membrane remodeling, enzyme maturation, and metabolic allocation.

Bioinformatic predictions, electrochemical measurements, redox cofactor analyses, and biofilm phenotypes in sum indicate that sRNAs modulate metabolism and surface behavior to enhance inward electron uptake and succinate production. To visualize this integrated framework, Figure 6 summarizes how polymer exposure activates sRNAs that reprogram gene expression and cellular metabolism to enhance bioelectrochemical performance. Experimental delivery of reconstructed sRNAs demonstrates that post‐transcriptional programs are sufficient to influence electron uptake and product formation, establishing sRNAs as a central regulatory axis linking material interfaces to bacterial metabolism and inward EET.

**FIGURE 6:**
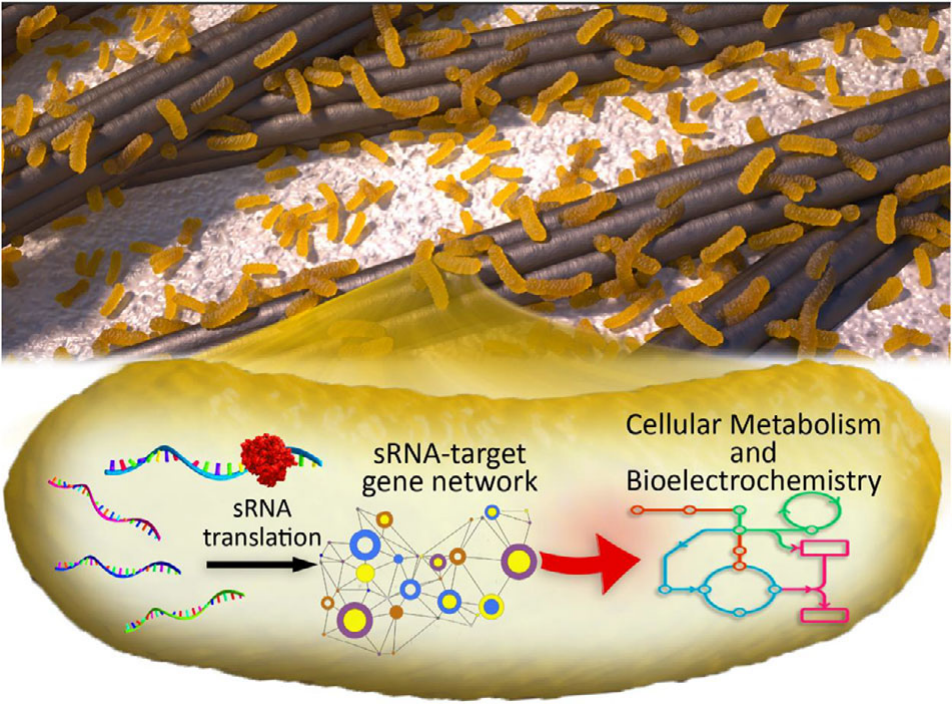
Schematic summary of the PBFDO–*S. oneidensis* interaction and its regulatory cascade. Polymer‐coated electrodes promote *S. oneidensis* adhesion and biofilm formation (top left), where PBFDO exposure triggers sRNA production within the bacterial cytoplasm. The zoom‐in illustrates how these sRNAs regulate target genes, influencing pathways linked to membrane composition, transport, and redox enzyme maturation. The resulting transcriptional and post‐transcriptional reprogramming enhances intracellular metabolism and extracellular electron transfer, depicted as cyclic arrows coupling redox flow and energy conversion.

## 3 Conclusion

Conjugated redox‐active polymers are widely assumed to increase microbial electron transfer through passive mechanisms such as increased interfacial conductivity, expanded electroactive surface area, or stabilization of diffusible redox mediators. Here, we show that conjugated polymers can also function as regulators of microbial physiology. Using the n‐type polymer PBFDO as a model system, we show that PBFDO‐coated electrodes operating under inward EET conditions are associated with coordinated regulatory and metabolic reprogramming in *S. oneidensis*, resulting in enhanced inward electron transfer and increased electrosynthetic output.

The overall response spans multiple biological levels. Electrochemical measurements reveal increased electron uptake and fumarate reduction, while transcriptomic and metabolomic analyses indicate altered intracellular redox and metabolic states, reinforcement of energy‐conserving pathways, and stabilization of a surface‐associated biofilm state. Partial substrate–product carbon accounting further demonstrated that PBFDO‐supported inward electron transfer increased both fumarate consumption and the fraction of consumed fumarate carbon recovered as succinate. These effects cannot be attributed solely to increased biomass or passive enhancement of electron transfer, as PBFDO‐supported reactors exhibit coordinated transcriptional, metabolic, post‐transcriptional, and protein‐level responses that are absent on carbon electrodes operated under identical electrochemical conditions. In particular, transcriptional responses associated with electron‐transfer pathways are accompanied by increased abundance of heme‐containing proteins, consistent with remodeling of cytochrome‐associated respiratory functions.

A central finding of this work is that small non‐coding RNAs mediate polymer‐induced physiological reprogramming. We identify a set of PBFDO‐responsive sRNAs predicted to target genes involved in transport, ion homeostasis, cell‐envelope remodeling, and redox enzyme maturation. Importantly, experimental delivery of individual sRNAs is sufficient to partially reproduce selected features of the polymer‐induced phenotype, including increased electron uptake and its downstream metabolic utilization, shifts in intracellular redox balance, and altered biofilm architecture. These findings establish sRNAs as post‐transcriptional modulators of electron transfer and metabolism, and support the conclusion that polymer exposure actively triggers sRNA‐mediated regulation.

The molecular mechanisms linking polymer surface properties to sRNA induction remain to be fully defined. One plausible explanation is that polymers such as PBFDO alter local redox buffering, surface charge, or envelope stress in ways that are sensed by the cell as a distinct environmental state. In this view, sRNAs act as rapid‐response regulators that translate interfacial cues into coordinated metabolic and structural adaptation. The integration of electrochemical, transcriptional, metabolic, and regulatory data supports a model in which PBFDO functions not only as an electron‐transfer material but also as a physiological modulator defined by the biotic/abiotic interface. Importantly, sRNA delivery is used here as a perturbation tool to test regulatory sufficiency, rather than to fully recapitulate endogenous polymer‐induced programs.

Beyond mechanistic insight, this work introduces sRNAs as a practical and underexplored control layer in microbial electrochemical systems. Compared with material redesign or permanent genetic modification, sRNA‐based perturbation offers a modular and reversible strategy for tuning microbial phenotype and electron‐transfer behavior. More broadly, these findings demonstrate that biohybrid interfaces can be engineered not only to transport charge but also to engage bacterial regulatory networks. This polymer–sRNA–metabolism axis opens new opportunities for controlling microbial electrosynthesis, energy conversion, and bioelectronic function through informed integration of materials chemistry and regulatory biology. Future work should define how specific surface chemistries and redox environments activate sRNA expression and harness this regulatory layer to program microbial electrosynthesis and bioelectronic technologies.

## Author Contributions

**David Ohayon**: writing – original draft, conceptualization, validation, writing – review and editing. **Yuanmei Liang**: conceptualization, investigation, writing – original draft, writing – review and editing, methodology, validation. **Zexuan Wang**: validation, writing – review and editing. **Jee Loon Foo**: methodology, writing – review and editing. **Glenn Quek**: methodology, writing – review and editing. **Samantha R. Mccuskey**: methodology, writing – review and editing. **Zhongxin Chen**: methodology, writing – review and editing. **Matthew Wook Chang**: funding acquisition, writing – original draft, writing – review and editing, validation, conceptualization. **Guillermo C. Bazan**: funding acquisition, writing – original draft, conceptualization, validation, writing – review and editing.

## Conflicts of Interest

The authors declare no conflicts of interest.

## Supporting information




**Supporting File 1**: advs77927‐sup‐0001‐SuppMat.docx.

**Supporting File 2**: advs77927‐sup‐0002TableS1.xlsx.

## Data Availability

Raw RNA‐seq data have been deposited in the NCBI Sequence Read Archive (SRA) under BioProject accession PRJNA1490096 (to be released upon publication). Processed count matrices and differential‐expression analyses are provided as Supplementary Data. Raw electrochemical datasets (chronoamperometry, cyclic voltammetry, and differential pulse voltammetry) are included in the Supporting Information. Additional source data, including metabolomics peak‐area tables, qPCR Ct values, and densitometric analyses of heme‐stained gels, are available from the corresponding authors upon reasonable request.
