# Supplementary material for: Conjugated Polymer‐Microbe Interactions Trigger Regulatory Reprogramming for Enhanced Bioelectrocatalysis
Source: Adv Sci (Weinh). 2026 Oct 4:e77927. Online ahead of print. doi: 10.1002/advs.77927 (PMC13634483; doi:10.1002/advs.77927)
Supplement: Supplementary file 1 [file ADVS-9999-e77927-s001.docx]

**Supplementary Information**

Conjugated Polymer-Microbe Interactions Trigger Regulatory Reprogramming for Enhanced Bioelectrocatalysis

David Ohayon,^1,†^ Yuanmei Liang,^2,3,4,5,†^, Zexuan Wang,^2,3,4,5^ Jee Loon Foo, ^2,3,4,5^ Glenn Quek,^6^ Samantha R. McCuskey,^7^ Zhongxin Chen,^1^ Matthew Wook Chang,^2,3,4,5^* Guillermo C. Bazan^1,7^*

^1^ Institute for Functional Intelligent Materials, National University of Singapore, Singapore 117544.

^2^ NUS Synthetic Biology for Clinical and Technological Innovation (SynCTI), National University of Singapore, Singapore.

^3^ Synthetic Biology Translational Research Program, Yong Loo Lin School of Medicine, National University of Singapore, Singapore.

^4^ Department of Biochemistry, Yong Loo Lin School of Medicine, National University of Singapore, Singapore.

^5^ National Centre for Engineering Biology (NCEB), Singapore.

^6^ Departments of Chemistry and Chemical & Biomolecular Engineering, National University of Singapore, Singapore 119077.

^7^ Institute for Digital Molecular Analytics and Science (IDMxS), Nanyang Technological University, Singapore, Singapore.

^†^ These authors contributed equally.

* Corresponding authors: [chmbgc@nus.edu.sg](mailto:chmbgc@nus.edu.sg); [bchcmw@nus.edu.sg](mailto:bchcmw@nus.edu.sg)

**Materials and Methods**

***Materials*.** H-BFDO precursor was purchased from Sagechem. Dimethyl sulfoxide (DMSO, Extra Dry, 99.7%), and duroquinone were purchased from Sigma. All chemicals were purchased from Fisher Scientific or Sigma Aldrich and used as received unless otherwise indicated.

***PBFDO synthesis.*** PBFDO was synthesized following the literature procedure.^1,2^ The PBFDO solution (1.83 mg/mL) in DMSO was then stored in the dark under ambient conditions.

*Microbial bioreactor preparation.* The 3-electrode single-chambered microbial bioreactors (15mL working volume) were assembled as follows. An Ag/AgCl (3.5 M KCl) served as the reference electrode, while counter and working electrodes were fabricated from 1 cm × 1 cm × 0.318 cm carbon felt (Alfa Aesar), woven with 0.25 mm Ti wire (Aldrich) as the electrical lead. For biohybrid bioreactors, the working electrode was modified with the PBFDO polymer: PBFDO was dropcasted (250 μL) from a 1.83 mg/mL solution in DMSO on an O_2_ plasma-activated carbon felt electrode and left to dry in a vacuum oven at 60 C° for at least 4h. Anaerobic conditions were maintained through constant headspace degassing with humidified, deoxygenated N_2_. Temperature was kept at 30 °C by housing the microbial bioreactors in a temperature-regulated incubator.

***Electrochemical characterization*.** All electrochemical measurements were conducted using a VMP300 BioLogic Potentiostat controlled with EC-Lab software. A three-electrode configuration was employed using carbon felt working and counter electrodes and an Ag/AgCl (3.5 M KCl) reference electrode. Unless otherwise stated, all measurements were performed under nitrogen-purged electrolyte/medium, and all potentials are reported versus Ag/AgCl (3.5 M KCl). Reactors were operated for an average of 60 h. To collect sufficient biomass for transcriptomic analysis, reactors were operated for an average of 120 h. Chronoamperometric measurements were performed at the potentials indicated in the corresponding figures. Following reactor operation, cyclic voltammetry (CV) and differential pulse voltammetry (DPV) were performed to characterize electrode-associated and soluble redox-active species. CV measurements were acquired at a scan rate of 5 mV/s. DPV measurements were conducted from −0.80 V to +0.20 V versus Ag/AgCl, using a quiescent time of 15 s, pulse height of 50 mV, pulse width of 300 ms, step height of 2 mV, and step time of 500 ms. To distinguish electrode-associated and soluble redox contributions, electrochemical measurements were performed under three conditions: (i) directly on colonized electrodes following current collection, (ii) on the same electrodes after rinsing and transfer into fresh degassed medium, and (iii) on spent supernatants analyzed using a clean glassy carbon working electrode. Identical CV and DPV parameters were used for all comparative measurements.

***Scanning-Electron Microscopy*.** After bioelectrochemical experiments, working electrodes were removed from the microbial bioreactor and gently rinsed with fresh n-SBM medium to remove non-attached cells. Electrodes were then immersed in a 2.5% glutaraldehyde in M1 medium fresh solution at 4°C overnight to chemically fix electrode-associated cells for 24 hours. Then, electrodes were sequentially rinsed by exchanging the medium with the following solutions for 20 minutes each: deionized water, 10, 30, 50, 70, and 100% ethanol, before being left to dry in ambient.

Working electrodes were sputtered with platinum (Anatech Hummer Sputtering System) before imaging with a Hitachi FlexSEM instrument. Accelerating voltage: 5 keV, working distance: ~6 mm, stage current: 50 pA, secondary electron detector.

***Fluorescence microscopy*.** Live/dead staining of cells was performed with 5 μM SYTO9, 20 minutes, 30 μM propidium iodide, 10 minutes, with 10 minutes rinsing in between each stain. Samples were viewed using a Leica Thunder fluorescence microscope and 40x objective. Images were processed using LasX software.

***Cell culture and growth medium.*** *Shewanella oneidensis* MR-1 (ATCC 700550) was struck out on LB agar plates from frozen bacterial stock and incubated at 30 °C to isolate single colonies. Liquid cultures were grown by selecting morphologically similar colonies with a sterile loop to inoculate anaerobic modified M1 medium (SBM) containing 20 mM Na-(L)-lactate as electron donor and 20 mM Na-fumarate as electron acceptor. SBM medium is a defined medium with the following composition per litre: 3 g HEPES buffer, 0.225 g potassium phosphate monobasic, 0.225 g potassium phosphate dibasic, 0.225 g ammonium sulphate, 0.46 g ammonium chloride, 0.117 g magnesium sulphate heptahydrate, 0.5 g casamino acids, 10 mL DL vitamin mix, 10 mL Wolfe’s mineral mix. After 24 hours incubation at 30 °C with shaking at 120 rpm, cultures consistently reach a maximum OD600 of ~0.3. These stationary phase cultures were then centrifuged, washed, and resuspended in modified SBM medium (without vitamin mix and casamino acids that can act as electron donors, -n-SBM) before being used to inoculate microbial bioreactors with an additional 50 mM of fumarate. Fumarate here functions as the sole metabolizable carbon substrate during electrochemical experiments. Following inoculation, cells were transferred to electrochemical reactors operated under identical medium conditions. Metabolomic analyses were further used to compare relative intracellular metabolite abundances between conditions and were not intended as quantitative carbon-flux measurements. Untargeted metabolomic analyses were used to compare relative intracellular metabolite abundances between conditions and were not intended to provide quantitative metabolic-flux or isotope-resolved carbon-tracing information.

***Cell culture and growth medium*.** *Shewanella oneidensis* MR-1 (ATCC 700550) was struck out on LB agar plates from frozen bacterial stock and incubated at 30 °C to isolate single colonies. Liquid cultures were grown by selecting morphologically similar colonies with a sterile loop to inoculate anaerobic modified M1 medium (SBM) containing 20 mM Na-(L)-lactate as electron donor and 20 mM Na-fumarate as electron acceptor. SBM medium is a defined medium with the following composition per litre: 3 g HEPES buffer, 0.225 g potassium phosphate monobasic, 0.225 g potassium phosphate dibasic, 0.225 g ammonium sulphate, 0.46 g ammonium chloride, 0.117 g magnesium sulphate heptahydrate, 0.5 g casamino acids, 10 mL DL vitamin mix, 10 mL Wolfe’s mineral mix. After 24 hours incubation at 30 °C with shaking at 120 rpm, cultures consistently reach a maximum OD600 of ~0.3. These stationary phase cultures were then centrifuged, washed, and resuspended in modified SBM medium (without vitamin mix and casamino acids that can act as electron donors, -n-SBM) before being used to inoculate microbial bioreactors with an additional 50 mM of fumarate. Fumarate here functions as the sole metabolizable carbon substrate during electrochemical experiments. Following inoculation, cells were transferred to electrochemical reactors operated under identical medium conditions. Metabolomic analyses were further used to compare relative intracellular metabolite abundances between conditions and were not intended as quantitative carbon-flux measurements. Untargeted metabolomic analyses were used to compare relative intracellular metabolite abundances between conditions and were not intended to provide quantitative metabolic-flux or isotope-resolved carbon-tracing information.

***Biomass quantification*.** Electrodes were gently rinsed with 100 mM PBS to remove planktonic cells and then placed in 15 mL tubes with 2 mL of 0.2 M NaOH and kept at 4 ˚C for 1 hour. Samples were vortexed for 10 seconds every 15 minutes. Electrodes were then rinsed with 2 mL of ultrapure water. To lyse the cells, samples were frozen at -20 ˚C then thawed at 90 ˚C for 10 minutes, repeated 3 times. 96-well plates were used to test the samples with the Coomassie Plus Reagent against bovine serum albumin standards made with 0.1 M NaOH diluent.

***Protein quantification.*** Heme-Stained SDS-PAGE Electrode-associated biofilms were harvested after current collection of bioreactors for at least 60h. Electrodes were rinsed in ice-cold 0.85% NaCl and lysed in ice-cold lysis buffer (50 mM Tris-HCl pH 7.5, 150 mM NaCl, 1 mM MgCl₂) by repeated vortexing and bath sonication, followed by probe sonication and centrifugation (3,000 × g, 2 min, 4°C) to remove debris. Protein concentration was determined by NanoDrop spectrophotometry, and 15 µg per lane was resolved on a 4–20% gradient SDS-PAGE gel under non-reducing, non-denaturing conditions. Heme-containing proteins were detected using TMBZ staining (6.3 mM TMBZ in methanol, 3:7 diluted in 0.25 M sodium acetate pH 5.0) developed with 30 mM H₂O₂. Band intensities were quantified using ImageJ software.

***High-performance liquid chromatography (HPLC) analysis*.** An aliquot was removed from the microbial bioreactors. HPLC analyses of succinate were performed using an Aminex HPX-87H column (Bio-rad) with the following parameters: 0.008 N H_2_SO_4_ mobile phase (0.6 mL min-1), 100 μL injection volume, column temperature of 35 °C and UV detection at 210 nm.

***Coulombic efficiency to succinate and electron partition index***

*Coulombic efficiency calculations*. Time-resolved current 𝑖(𝑡) was integrated over 40h to obtain the total charge:

$$Q = \int i(t) dt$$

Electron moles from the electrode were computed as:

$$n\_e = Q / F$$

with F = 96485 C mol⁻¹ the Faraday constant.

For the succinate quantification and electron equivalents, the endpoint succinate concentration (C_succ (mM)) was measured by HPLC and converted to moles and electron equivalents:

$$n_{\mathrm{succ}}= C_{\mathrm{succ}}\times{10}^{-3}\times V \left( L \right),n\_e,succ = 2 \times n\_succ$$

reflecting the 2-electron stoichiometry of fumarate to succinate reaction.

To isolate the contribution of PBFDO, “net” quantities were defined by subtraction (PBFDO − Control):

$$\Delta Q = Q_{\mathrm{PBFDO}}- Q_{\mathrm{Ctrl}}, \Delta n_{e}= n_{e},PBFDO - n_{e},Ctrl，$$

$$\Delta n\_e,succ = n\_e,succ,PBFDO- n\_e,succ,C\mathrm{trl}$$

Two metrics were reported:

(1) Coulombic efficiency to succinate (net):

$$CE\_succ,net (\%) = (\Delta n\_e,succ / \Delta n\_e) \times100\%$$

(2) Electron Partition Index (EPI):

$$EPI = \Delta n\_e / \Delta n\_e,succ$$

***RNA extraction, purification, and characterization*** *(electrode-attached biofilms*. At each sampling time, working electrodes were gently rinsed with sterile 1× PBS to remove loosely associated medium, blotted on sterile lint-free wipes, and immediately submerged in RNAlater (Thermo Fisher; ≥10 µL RNAlater per mg wet biomass). Electrodes were held at 4 °C for 12–24 h to allow penetration and then stored at −80 °C until extraction.

For lysis, each electrode was transferred to a 2 mL screw-cap tube preloaded with ~0.5 mL acid-washed 0.1 mm zirconia/silica beads and 1.0 mL TRIzol Reagent (Invitrogen). Cells were disrupted in a bead beater (FastPrep-24, MP Biomedicals) for 2 × 30 s at 6.0–6.5 m/s with 2 min on ice between pulses. Lysates were clarified (12,000 ×g, 10 min, 4 °C) and transferred to RNase-free tubes. Phase separation was performed by adding 200 µL chloroform per 1.0 mL TRIzol, vigorous shaking for 15 s, incubation for 3 min at room temperature, and centrifugation (12,000 ×g, 15 min, 4 °C). The aqueous phase was recovered, mixed 1:1 with 70% ethanol, and applied to a silica spin column compatible with TRIzol lysates (e.g., Direct-zol RNA MiniPrep, Zymo Research). On-column DNase I treatment (15 min) was included to remove genomic DNA. Columns were washed and RNA was eluted in 30–50 µL nuclease-free water and kept on ice. For PBFDO-coated electrodes, to minimize hydrophobic polymer carryover, a Phase Lock Gel tube was used during chloroform partitioning or an additional chloroform back-extraction of the organic phase was performed prior to column binding.

RNA concentration and purity were assessed by NanoDrop (A260/280 ≈ 2.0; A260/230 ≥ 1.8) and Qubit RNA HS assays. Integrity was evaluated on an Agilent 2100 Bioanalyzer (RNA 6000 Pico) or TapeStation; samples with RIN/RQI ≥ 7.5 were accepted. Residual DNA was checked by no-RT qPCR targeting a housekeeping locus; any positives were re-treated with DNase. RNA-seq libraries were prepared from approximately equal total RNA inputs, and differential expression analysis was performed using DESeq2 with estimated size-factor normalization. This approach compares transcriptome composition on an approximately equivalent total-RNA basis and does not directly report transcript abundance per cell.

***Library preparation and sequencing*** *(SMART-Seq Total RNA Mid Input with UDI 1–24)*. DNA-free RNA (typically 10–100 ng per sample) was used to prepare strand-specific total-RNA libraries with the SMART-Seq Total RNA Mid Input Kit (Takara Bio), following the manufacturer’s instructions. Briefly, cDNA was synthesized via SMART template switching, rRNA signals were removed according to the kit workflow, and strand-specific libraries were constructed and amplified with 12–15 PCR cycles (cycle number optimized by pre-library qPCR). Unique dual indexes were applied using the Takara Unique Dual Index (UDI) Kit, set 1–24, assigning a distinct i7/i5 pair to each sample.

Post-PCR libraries were purified with AMPure XP beads (typically 0.9× ratio), quantified by Qubit dsDNA HS assay, and assessed on an Agilent Bioanalyzer High Sensitivity DNA kit to confirm the expected insert size distribution (~300–450 bp; total library size ~400–600 bp) and absence of primer dimers. Equimolar pooling was performed to a final 2–4 nM pool. Pooled libraries were shipped on dry ice to Novogene (Singapore) and sequenced on an Illumina platform (e.g., NovaSeq 6000) with paired-end 150 bp (PE150) reads, targeting ≥ 10–20 million read pairs per sample. Novogene performed inbound QC (library concentration, size profile, residual rRNA fraction); only libraries passing all thresholds were included in downstream analyses. For comparability with electrochemical measurements, matched aliquots were taken for cell counting (CFU or flow cytometry) at sampling, enabling per-cell normalization in transcriptomic/electrochemical integration analyses.

***Computational identification of PBFDO-responsive sRNAs*.** Analyses used stranded, rRNA-depleted total-RNA data; therefore sRNAs were discovered from coverage features. For QC and alignment, adapters and low-quality bases were trimmed with fastp v0.23. Clean reads were aligned to the *S. oneidensis* reference genome with bowtie2 v2.5 (very-sensitive, stranded RF). BAMs were sorted/indexed (samtools v1.18). Per-strand coverage bedGraphs/bigWigs were produced with bedtools genomecov v2.31, masking rRNA/tRNA/repeats.

For candidate extraction (30-300 nt), continuous coverage “islands” were scanned in intergenic regions and antisense to annotated ORFs. Segments of 30–300 nt with mean coverage above the replicate-specific 25th percentile of expressed genes were retained; overlapping segments were merged. Putative 5′/3′-UTR extensions (sense to neighboring ORFs without an independent promoter) were removed.

Secondary structure at 37 °C was predicted with RNAfold (ViennaRNA v2.6); candidates with stable hairpins (e.g., ΔG ≤ −12 kcal mol⁻¹ or top-quartile stability) were prioritized. Upstream σ⁷⁰-like −35/−10 motifs were identified with MEME/FIMO v5.5 (spacing 15–19 bp; p < 1e−4). Downstream Rho-independent terminators were called by TransTermHP v2.09 (hairpin followed by ≥ 4 U; terminator ΔG ≤ −8 kcal mol⁻¹). The presence of both promoter and terminator supported an independent transcription unit.

Strand-aware counts for candidate sRNAs were obtained with featureCounts (Subread v2.0) using custom SAF annotations. Differential expression (PBFDO *vs*. control electrodes) was tested with DESeq2 v1.40; candidates with |log₂FC| ≥ 1 and FDR < 0.05 (consistent across replicates) were designated PBFDO-responsive and assigned identifiers (sRNA_001, … ,Table S3).

***Target prediction & functional assignment*.** IntaRNA v3.3 (TargetRNA2 as cross-check) was run against 5′-UTR/RBS windows (−60 to +30 nt relative to the start codon) of genes in pathways relevant to extracellular electron transfer—particularly ccmABCDEFGH (cytochrome-c maturation) and mtr/omc (outer-membrane multi-heme cytochromes). Interactions were ranked by ΔG, seed length 7–12 nt, site position near the RBS, and conservation across Shewanella spp. Candidates were mapped to functional modules (EET, cell envelope, ion homeostasis, metal uptake) based on KEGG/MetaCyc annotations. All loci and interactions were reviewed in IGV v2.16 using strand-specific coverage tracks.

*Plasmid preparation and strain construction.* The sRNA sequences were amplified from *S. oneidensis* MR-1 genomic DNA and cloned into pBbE8K via Gibson assembly, yielding plasmids pB-S1, pB-S2, pB-S3, pB-S4, and pB-S5. The empty pBbE8K vector and these sRNA plasmids were introduced into *S. oneidensis* MR-1 by electroporation to generate the control strain (Ctrl) and the sRNA-expressing strains S1, S2, S3, S4, and S5, respectively.

*Untargeted metabolomics by HILIC (Agilent 1290–6550 Q-TOF)*. Working electrodes were briefly rinsed in ice-cold 1× PBS, blotted, and immediately submerged in methanol:acetonitrile:water (40:40:20, v/v/v, −20 °C) containing internal standards (Benzoic acid, 2µM). Samples were transferred to 2 mL screw-cap tubes with 0.5 mL of 0.1 mm zirconia/silica beads and lysed on a FastPrep-24 (6.0–6.5 m/s, 30 s ×2, 2 min on ice between pulses). After a 10 min hold at −20 °C, lysates were centrifuged (15,000×g, 10 min, 4 °C); supernatants were collected. Pellets were re-extracted with 0.5 mL of the same solvent, pooled, dried (vacuum concentrator, ≤30 °C), and stored at −80 °C. For Liquid Chromatography-Mass Spectrometry (LC–MS), residues were reconstituted in 80% acetonitrile at ~10 µL per 10⁸ cells (or protein-normalized), vortexed, sonicated on ice (2 min), clarified (15,000×g, 5 min, 4 °C), and transferred to glass-insert vials. A pooled QC (equal aliquots of all samples) and solvent blanks were prepared.

***Ultra High-Performance Liquid Chromatography (UHPLC) with Hydrophilic Interaction Liquid Chromatography (HILIC)***. Analyses used an Agilent 1290 Infinity/II UHPLC (autosampler 4 °C; injection 3–5 µL) with an InfinityLab Poroshell 120 HILIC-Z, 2.1×100 mm, 2.7 µm column at 35 °C. Mobile phase A (ESI+) was 10 mM ammonium formate + 0.1% formic acid in water (for ESI−: 10 mM ammonium acetate, pH 9.0); B was acetonitrile. Flow 0.30 mL/min. Gradient (%B vs min): 0/90, 2/90, 10/60, 12/40, 14/40, 15/90, 20/90 (re-equilibration).

***Q-TOF MS (Jet Stream ESI****)*. Data were acquired on an Agilent 6550 iFunnel Q-TOF in centroid mode over m/z 50–1,100 in both polarities. Typical source settings (optimized on pooled QC): gas temp 200–250 °C; drying gas 12–14 L/min; nebulizer 35–45 psig; sheath gas 350 °C, 10–12 L/min; VCap +3.5 kV/−3.0 kV; fragmentor ~120 V. Lock-mass ions were infused continuously: ESI+ m/z 121.050873 & 922.009798; ESI− m/z 112.985587 & 1033.988109. MS¹ rate ~5 Hz. AutoMS/MS (optional): Top-5, isolation ~1.3 m/z, dynamic **exclusion 0.3–0.5 min, stepped CE 10/20/40 eV.**

***Processing and statistics*.** Vendor .d files were processed in MassHunter Profinder (MFE/BMFE) or converted to .mzML and processed in MS-DIAL/MZmine for peak detection, alignment, adduct/isotope grouping (tolerances: ≤10 ppm, ≤0.20 min RT). Features with blank/mean ≥ 0.5 were removed. QC-based LOESS (QC-RLSC) was used for drift correction; normalization proceeded internal-standard ratio (if spiked) → TIC or PQN → per-cell or per-protein. MS/MS matching to METLIN, HMDB, NIST, MoNA/GNPS supported annotation; identifications were assigned MSI levels (key metabolites Level 1–2). Statistics used Welch’s t-test or linear mixed-effects models (for time×treatment), with Benjamini–Hochberg FDR control (q<0.05); PCA/PLS-DA were cross-validated.

***Reverse Transcription Quantitative PCR (RT-qPCR)***. DNase-treated total RNA (electrode-attached biofilms; RIN/RQI ≥7.5) was reverse-transcribed with HiScript III All-in-one RT SuperMix Perfect for qPCR (Vazyme) per manufacturer instructions (≈500 ng RNA per 20 µL; cDNA diluted 1:5–1:10). qPCR used Luna® Universal qPCR Master Mix (2×) (NEB) in 20 µL with 0.2 µM primers (Table S2) on a real-time thermocycler (95 °C 60 s; 40×: 95 °C 15 s, 60 °C 30 s), followed by a melt curve to confirm single products. Primers (80–175 bp amplicons, Tm ≈ 60 °C) were validated for efficiency (90–110%) and specificity; no-RT and no-template controls were included. Genes for qPCR validation were selected based on their biological relevance to extracellular electron transfer (EET), energy metabolism, and biofilm formation, rather than solely on RNA-seq significance thresholds, in order to provide independent confirmation of transcriptional trends across functionally important categories(Table S1b (Supplementary File). Relative transcript abundance was calculated using the 2^−ΔΔCt method with normalization to the geometric mean of the housekeeping genes *gyrB* and *rpoA*. These reference genes were selected based on their established use as stable housekeeping genes in *S. oneidensis* MR-1 and because neither gene exhibited significant differential expression in our RNA-seq dataset (gyrB: log₂FC = 0.89, padj = 0.23; rpoA: log₂FC = 0.18, padj = 0.998) under the experimental conditions used in this study.^3,4^ Biological replicates (n ≥ 3) with technical triplicates were analyzed, and only assays exhibiting a single melt peak, no amplification in no-template controls, and consistent technical replicates were included.

***Quantification of the intracellular NAD^+^/NADH.*** NAD^+^/NADH concentrations were measured using the Sigma-Aldrich NAD/NADH Assay Kit according to manufacturer’s instructions. *Shewanella oneidensis* MR-1 strains under anaerobic conditions were sampled for NAD(H) quantification. Cells from working electrodes were collected, washed with cold PBS, and resuspended in 0.5 mL of NAD Extraction Buffer for NAD^+^ determination or in NADH Extraction Buffer for NADH determination. The cells were then lysed by ultrasonication on an ice bath using an Ultrasonic Homogenizer. The cell lysates were further boiled for 5 min, then 20 µL of Assay Buffer and 100 µL of the opposite extraction buffer were added to neutralize the extracts. The samples were cooled on an ice bath and centrifuged at 13,000 × g and 4°C for 10 min to remove cell debris. A 200 µL aliquot of each supernatant was transferred into separate wells of a clear-bottom 96-well plate for NAD^+^/NADH assays.

**Supplementary Discussion S1: Extended Interpretation of Transcriptomic and Metabolic Responses to PBFDO**

**S1.1. Respiratory network context and polymer-specific effects**

The canonical respiratory architecture of Shewanella oneidensis links the inner-membrane quinone pool to extracellular electron acceptors through the CymA-centered periplasmic network and the outer-membrane MtrCAB conduit.^5-7^ While we emphasize pathway-level coordination in the manuscript, we here provide additional mechanistic context for how polymer exposure may reshape electron routing within this network.

Transcriptomic analysis revealed coordinated modulation of genes spanning inner-membrane electron entry (cymA), periplasmic electron sinks (fccA), and downstream energy-conversion modules (nuo, atp). Importantly, this response does not reflect uniform upregulation of all EET-related components. Instead, downregulation of bacterioferritin (bfr) suggests reduced sequestration of intracellular iron, potentially mobilizing iron cofactors toward cytochrome biogenesis and expanding the respiratory electron-transfer capacity. Concomitant downregulation of the iron hydrogenase large subunit (hydA) further indicates reduced flux toward competing electron sinks, consistent with preferential routing of electrode-derived electrons into the CymA–FccA periplasmic pathway. This selective remodeling supports the interpretation that PBFDO stabilizes electron transfer through altered electron partitioning rather than uniform upscaling of existing pathways.

**S1.2. Inner-membrane energy-conversion modules and redox balancing**

Upregulation of Complex I subunits (nuoF, nuoL) and ATP synthase (atpD) suggests enhanced coupling between electron influx and proton-motive force (PMF) generation. NuoF contains the FMN-binding site that initiates electron transfer from NADH into the membrane arm of Complex I, while NuoL participates in proton translocation.^8-10^ Co-induction of these subunits implies that polymer-mediated electron uptake supports increased respiratory turnover rather than passive accumulation of reduced quinones.

**S1.3. Interpretation of fccA induction and periplasmic electron sinks**

The co-induction of fccA with conduit and energy-conversion genes suggests reinforcement of periplasmic electron sinks under PBFDO exposure. FccA accepts electrons from the CymA-linked periplasmic network and catalyzes fumarate reduction to succinate, a dominant inward EET pathway in S. oneidensis.^7^

However, transcriptional upregulation of fccA alone does not establish whether fumarate reduction becomes the dominant electron sink or functions as a buffering pathway to accommodate increased electron flux. Because the periplasmic network feeds multiple terminal reductases, transcriptional data must be interpreted in the context of metabolic allocation and charge balance. This uncertainty motivates the combined use of Coulombic efficiency (CE) and Electron Partition Index (EPI) metrics in the metabolomic analysis.

**S1.4. Electron partitioning: CE, EPI, and physiological electron use**

Product-based CE captures only the fraction of electrons recovered in a defined, measurable product (here, succinate). In bioelectrochemical systems, this metric often underestimates physiological electron utilization, as electrons can be diverted into intracellular redox pools, maintenance energy, and biomass-associated processes.^11^

To address this limitation, we define the EPI as the ratio of total electrons transferred from the electrode to the number of electrons recovered in succinate. An EPI greater than unity indicates substantial allocation of electrons beyond the measured product sink. In PBFDO systems, an EPI of ~3.4 indicates that roughly one-third of additional electrons are recovered in succinate, while the remainder contribute to redox and energy conservation, consistent with elevated NADH/NAD⁺ and ATP/ADP ratios.

This framework reconciles increased succinate production with broader metabolic activation and supports the conclusion that PBFDO enhances total inward EET throughput rather than selectively funneling electrons into a single metabolic pathway.

**S1.5. Polymer versus bare electrode: why the interface matters**

Both PBFDO-coated and bare carbon electrodes impose inward EET conditions; therefore, differences in transcriptional and metabolic states cannot be attributed to electron uptake per se. Instead, polymer-specific effects likely arise from altered interfacial kinetics and redox buffering.

PBFDO introduces a soft, redox-active matrix that lowers charge-transfer resistance, increases effective electronic contact area, and provides pseudocapacitive buffering at the biofilm–electrode interface.^12^ These properties likely stabilize electron delivery into the quinone pool and periplasmic network, reducing stochastic fluctuations in electron availability that occur on bare electrodes.

Endogenous flavins (riboflavin and FMN), which are secreted by S. oneidensis and adsorb at electrode surfaces, further mediate electron exchange. PBFDO likely synergizes with this native flavin layer by retaining redox mediators and facilitating electron hopping between polymer, flavins, and cytochromes (**Figure S6**). This synergy provides a mechanistic explanation for why polymer exposure reshapes downstream metabolic allocation even under identical electrochemical driving forces.

**S1.6. Adhesion, structure, and rate limitation**

Upregulation of the bpfA/bpfG/bpfD operon and increased biomass accumulation indicate that PBFDO enhances biofilm integrity and interfacial stability.61 However, normalization of current to biomass shows that increased adhesion alone cannot account for the observed gains in electron transfer. Instead, adhesion appears to play a permissive role by stabilizing cell–polymer contact, while transcriptional amplification of the Mtr–CymA axis and energy-conversion modules defines the dominant rate-limiting contribution.

This interpretation aligns with biophysical evidence that MtrCAB functions as a surface-exposed decaheme conduit capable of sustaining high electron flux when interfacial conditions are favorable.^13^

**Figure S1.** Comparison of HPLC traces obtained after current collection for the PBFDO-*S. oneidensis* biocomposite (dark blue), bacteria control (orange), and PBFDO control (light blue). PBFDO biocomposites exhibit a 12-fold enhancement in succinate accumulation relative to uncoated controls, confirming efficient inward extracellular electron transfer and metabolic coupling.

**Figure S2. a.** Total biomass extracted from PBFDO-coated and control electrodes after reactor operation. Biomass was determined by Bradford assay, showing approximately 5–10× higher biofilm density on PBFDO electrodes; **b**. Total DNA from PBFDO-coated and control electrodes after reactor operation.

**Figure S3. a**. Representative SEM images of *S. oneidensis* biofilms grown on PBFDO-coated carbon felt electrodes following cathodic operation at different potentials (-0.4, -0.6, and -0.8V *vs.* Ag/AgCl); **b**. Representative SEM images of *S. oneidensis* biofilms grown on PBFDO-coated carbon felt electrodes (left) and on carbon felt control electrodes (right), following cathodic operation at -0.8V *vs.* Ag/AgCl.

**Figure S4**. (**a**–**c**) Cyclic voltammograms and (**d**–**f**) differential pulse voltammograms obtained from bioelectrochemical reactors after current collection. Measurements were performed directly on colonized electrodes immediately after operation (**a**, **d**), on the same electrodes following rinsing and transfer into fresh degassed medium (**b**, **e**), and on spent supernatants using a clean glassy carbon electrode (**c**, **f**). Green traces correspond to PBFDO-coated electrodes colonized by *S. oneidensis*, grey traces to carbon-felt electrodes colonized by *S. oneidensis*, and orange traces to polymer-only controls, where indicated. Insets in each panel show the corresponding electrochemical response of the carbon-felt control on an expanded current scale to facilitate comparison of redox features. DPV traces of control bioreactors in **Figure S4e**, **f** were smoothed using an Adjacent Averaging (10 points) method.

Rinsing largely removes the broad catalytic response observed after operation, whereas catalytic activity is recovered in spent medium, indicating contributions from soluble redox-active species. DPV measurements reveal electrode-associated redox features consistent with outer-membrane cytochromes and flavin-related species, while a common soluble redox signal near −0.62 V vs Ag/AgCl is observed in supernatants from both conditions, suggesting a bacterial origin rather than polymer leaching.

**Figure S5.** Confocal fluorescence imaging of live/*dead* cell staining in electrode-associated biofilms. Representative confocal fluorescence images of *S. oneidensis* MR-1 biofilms stained with SYTO 9 (green; all cells) and propidium iodide (PI; red; membrane-compromised/dead cells) following chronoamperometric operation at −0.6 V vs. Ag/AgCl. Images are shown for bare carbon felt electrodes (no cells; autofluorescence control), carbon felt controls (Ctrl), and PBFDO-coated electrodes (PBFDO). Each panel represents a maximum intensity projection over the middle 25 focal planes of a confocal z-stack. Quantitative image analysis indicated 99.2% viable cells in control biofilms (0.8% PI-positive) and 93.5% viable cells in PBFDO-supported biofilms (6.5% PI-positive), demonstrating that neither the applied cathodic potential nor polymer exposure causes significant cytotoxicity. Scale bars, 100 µm.

**Figure S6.** Normalized flavin under (PBFDO vs Control). Data are presented as normalized values relative to the control group and shown as mean ± SD $\left( n = 3 \right.$biological replicates).

**Figure S7.** Bioinformatic pipeline for identification and functional prediction of PBFDO-responsive small RNAs (sRNAs). Workflow combining read assembly, secondary-structure modeling, target prediction (IntaRNA), and gene ontology classification of predicted mRNA targets.

**Figure S8.** Representative SEM images of biofilms grown on carbon felt electrodes following experimental introduction of sRNA_i constructs (labeled S1–S5) versus wild type (WT). Distinct biofilm morphologies correspond to sequence-dependent regulatory effects on surface colonization.

**Table S1. Differentially expressed genes (DEGs) and RT-qPCR validation data for *S. oneidensis* MR-1 under PBFDO exposure relative to carbon-electrode controls.**

**Table S1a**. All statistically significant DEGs identified by RNA-sequencing analysis of *S. oneidensis* MR-1 biofilms grown on PBFDO-coated versus uncoated carbon electrodes under inward extracellular electron transfer conditions. The table includes normalized expression values for each biological replicate (PBFDO1–3 and Ctrl1–3), gene annotations, predicted transcription-factor family associations, and functional classifications relevant to electron transfer, metabolism, membrane remodeling, transport, and stress adaptation. Differential expression was determined using padj < 0.05 and |log₂FC| > 1. Genes marked with ★ were selected for RT-qPCR validation.

**Table S1b**. RNA-seq expression statistics (log₂FC and padj) for all 13 RT-qPCR-validated genes, including those below the DEG significance threshold. Genes were selected for validation based on biological relevance to EET, energy metabolism, and biofilm formation, independent of RNA-seq significance status. All locus tags are referenced against the NCBI RefSeq annotation for *S. oneidensis* MR-1 (GCF_000146165.2).

For clarity and readability, this large dataset is provided as a separate supplementary file.

**Table S2. Primer sequence used for qPCR.**

| qPCR primers | Sequence (5’-3’) |
| --- | --- |
| ccmA-F | TGATACATCTGCCATATTGGC |
| ccmA-R | GCCAGAGTCTTGCCAATGCT |
| CymA-F | CACTTACCCCATGGCCCTGT |
| CymA-R | TCGTCTAACCAAGCTTGAGT |
| fccA-F | AACCGGTGGTGCAGGTGTTG |
| fccA-R | CTTCAAGGATGCGCACAACG |
| frdA -F | CATGACCGAGCATATGCGTA |
| frdA -R | CACGCAGGTTGGTTTCGATA |
| mtrB-F | GCGTTAGTGACGGATAACTA |
| mtrB-R | TTGCTCACTTTGCTAACGAC |
| mtrC-F | CGGCAATGATGGTAGTGATG |
| mtrC-R | GCTTCGTTAGTGGCGAAAAC |
| omcA-F | GGATACGGCGTTGAAGATGT |
| omcA-R | TGGTATCCGTTCCATTCCAT |
| atpD-F | GCGTTCTCGACAAAGTAGCC |
| atpD-R | TTCGTCACGGAATTTCTCAG |
| nuoF-F | GCAAACCCACCTGCGTTAAC |
| nuoF-R | GTGATCTTCTGAGCCCGGTA |
| pntA-F | TCTGATGAGTTTATCGCTGC |
| pntA-R | ATAAGTTTAGGTGCAGGACG |
| bpfA-F | CCCACTGCACCGACCGTATT |
| bpfA-R | ATGCTCACGGTCAACTGTAC |
| bpfG-F | GTACCCGAAGATAAGATGCG |
| bpfG-R | CTGTAAACGGGGATCAGATC |
| bpfD-F | TGGCGGATCATGTCCAACAC |
| bpfD-R | GTGCTAGGGAGTGGCATATC |
| gyrB-F | GGTAAGATTGTCGATGCT |
| gyrB-R | TCGGCCAGCTTTCCTGGCAA |
| rpoA-F | TATCAGCATGCGTATTCGCG |
| rpoA-R | TCAACACGAGCTGCTTCTAC |

**Table S3 Predicted sRNA information from IntaRNA analysis.**

| Name | sequence | MFE |
| --- | --- | --- |
| sRNA1 | TTATCGAAGTGCTAAAACGCCTCGAAGACGATGAAACCTTATTGGTGCAATCGGGCAAAC  CTGTGGGCGTATTTCGCACCCACGCGGATGCCCCTCGCGTGTTAATCGCTAACTCAAACTTAGTGCCACATTGGGCAAATTGGGAGCACTT | -45.7 |
| sRNA2 | TGTTGTAACAGTGCTATTGCTGCTTCAATTGCCATAACACGAATTTCATCGTGACTATGC  TCTTTGCGTCTCGCCATAGGTCTCACTCCCTGTTGAATGAGGTGAGATTAACAGCCAAAA  AAATAAAAAACAACGTTCAAAATAAG | -27.5 |
| sRNA3 | AGCAACCGCGCTGGAAAAAAGCGGTGGATGGTGCAGATACTGTGATTGGTGAGCTGGTGG  GAGAGGAGTATGTAAAACAATACTTCAAACCAGAAGCCAAAGCGCGCATGGAAACCATGA  TCAAAAATCTGATCAAAGGCTTTGAAGTCAGTATTAATGAACTGGAATGGATGACGCCAG  AGACTAAAGTT | -47.2 |
| sRNA4 | CAGCGGGTCGCGATAGCCAGGGCATTAGCCCACAATCCGAGTTTACTGCTGGCCGACGAA  CCCACAGGTAACTTAGATGACGAATCTGGTAAACAGGTGATGTCACTACTATGCGAACTT  G | -37.6 |
| sRNA5 | GCATCCATGCCGCCAAATTTGGCTCGCCATTTGTAAAAAGTCGCTGAGCTCATGCCATGT  TCGCGGCACAATTCTGGGACTGGAGTTCCGGCTTCAGCTTGTTTAAGGATTGCCATGATT  TGGCTATCGGTGAATTTTGAT | -41.2 |

**References**

1	Tang, H. *et al.* A solution-processed n-type conducting polymer with ultrahigh conductivity. *Nature* **611**, 271-277 (2022). <https://doi.org/10.1038/s41586-022-05295-8>

2	Ohayon, D. *et al.* High-Performance Aqueous Supercapacitors Based on a Self-Doped n-Type Conducting Polymer. *Advanced Materials* **36**, 2410512 (2024). <https://doi.org/https://doi.org/10.1002/adma.202410512>

3	Edel, M. *et al.* Extracellular riboflavin induces anaerobic biofilm formation in Shewanella oneidensis. *Biotechnology for Biofuels* **14**, 130 (2021). <https://doi.org/10.1186/s13068-021-01981-3>

4	Chen, Y., Cheng, M., Song, H. & Cao, Y. Type I-F CRISPR-PAIR platform for multi-mode regulation to boost extracellular electron transfer in Shewanella oneidensis. *iScience* **25**, 104491 (2022). <https://doi.org/https://doi.org/10.1016/j.isci.2022.104491>

5	Sturm, G. *et al.* A dynamic periplasmic electron transfer network enables respiratory flexibility beyond a thermodynamic regulatory regime. *The ISME Journal* **9**, 1802-1811 (2015). <https://doi.org/10.1038/ismej.2014.264>

6	Edwards, M. J., White, G. F., Butt, J. N., Richardson, D. J. & Clarke, T. A. The Crystal Structure of a Biological Insulated Transmembrane Molecular Wire. *Cell* **181**, 665-673.e610 (2020). <https://doi.org/https://doi.org/10.1016/j.cell.2020.03.032>

7	Alves, M. N. *et al.* Characterization of the periplasmic redox network that sustains the versatile anaerobic metabolism of Shewanella oneidensis MR-1. *Frontiers in Microbiology* **Volume 6 - 2015** (2015). <https://doi.org/10.3389/fmicb.2015.00665>

8	Steimle, S. *et al.* Role of Subunit NuoL for Proton Translocation by Respiratory Complex I. *Biochemistry* **50**, 3386-3393 (2011). <https://doi.org/10.1021/bi200264q>

9	Parey, K. *et al.* High-resolution structure and dynamics of mitochondrial complex I—Insights into the proton pumping mechanism. *Science Advances* **7**, eabj3221 (2021). <https://doi.org/doi:10.1126/sciadv.abj3221>

10	Friedrich, T. & Böttcher, B. The gross structure of the respiratory complex I: a Lego System. *Biochimica et Biophysica Acta (BBA) - Bioenergetics* **1608**, 1-9 (2004). <https://doi.org/https://doi.org/10.1016/j.bbabio.2003.10.002>

11	Im, C. *et al.* Low electric current in a bioelectrochemical system facilitates ethanol production from CO using CO-enriched mixed culture. *Frontiers in Microbiology* **Volume 15 - 2024** (2024). <https://doi.org/10.3389/fmicb.2024.1438758>

12	Zhu, Y. *et al.* Highly conductive PBFDO network bridging biofilm and electrode interface for electron capture and transfer in microbial electrochemical system. *Chemical Engineering Journal* **520**, 166160 (2025). <https://doi.org/https://doi.org/10.1016/j.cej.2025.166160>

13	Clarke, T. A. *et al.* Structure of a bacterial cell surface decaheme electron conduit. *Proceedings of the National Academy of Sciences* **108**, 9384-9389 (2011). <https://doi.org/doi:10.1073/pnas.1017200108>
